# Microbiota-derived 10-hydroxystearic acid activates PPARα to restore gut epithelial barrier integrity and enhance anti-retroviral therapy

**DOI:** 10.1038/s41564-026-02433-0

**Published:** 2026-07-30

**Authors:** Dylan Kramer, Clarissa Santos Rocha, Christopher A. Gaulke, Marie Nearing, Sumathi Sankaran-Walters, Ikaika Loque, Anugraha Kidigannappa, Eric Pham, Shuang Hu, Patrawin Wanakumjorn, Ramona Abbattista, Abhaya Dandekar, Roland Faller, Satya Dandekar

**Affiliations:** 1 Department of Medical Microbiology and Immunology, School of Medicine, University of California Davis, Davis, CA USA; 2 Department of Pathobiology, College of Veterinary Medicine, University of Illinois Urbana-Champaign, Champaign, IL USA; 3 Department of Materials Science and Engineering, University of California Davis, Davis, CA USA; 4 Department of Plant Sciences, University of California Davis, Davis, CA USA; 5 Department of Chemical Engineering, Texas Tech University, Lubbock, TX USA; 6 National Biomedical Research Institute at UC Davis, University of California Davis, Davis, CA USA

**Keywords:** Retrovirus, HIV infections

## Abstract

HIV infection disrupts gut epithelial barrier integrity and mucosal immunity, driving chronic inflammation and disease progression which are not fully resolved despite anti-retroviral therapy. Here we identify the microbiota-derived octadecanoid-hydroxy-fatty-acid metabolite 10-hydroxystearic acid (10-HSA), produced by *Lactiplantibacillus plantarum*, as a key mediator of gut epithelial barrier repair in human intestinal epithelial cells in vitro, ex vivo and in the non-human primate model of HIV/AIDS. X-ray crystallography and transcriptomics combined with functional analyses revealed that 10-HSA directly binds PPARα, inducing lipid metabolism, mitochondrial regeneration and subsequent epigenetic histone crotonylation, thereby promoting gut epithelial renewal. Co-administration of 10-HSA with anti-retroviral therapy in SIV-infected macaques accelerated viral suppression, resolved systemic inflammation, repaired gut epithelial integrity and recovered the gut microbiota. These findings identify a microbiota-derived lipid metabolite–PPARα–histone crotonylation axis that activates gut epithelial regeneration. This study defines a host–microbiome metabolic pathway that restores epithelial–immune homeostasis and enhances the efficacy of anti-retroviral therapy.

## Abstract (web summary)

*Lactiplantibacillus plantarum*-derived fatty-acid metabolite, 10-hydroxystearic acid, interacts directly with PPARα to alter histone crotonylation and the transcriptional landscape which leads to gut barrier functional repair and enhanced viral suppression in non-human primate models when combined with anti-retroviral therapy.

## Main

Chronic inflammation disrupts the gut microenvironment and impairs mucosal immunity^1,2^. Current understanding of gut mucosal host–microbe interactions has focused on microbial community composition, short-chain fatty acid (SCFA) production and alterations in amino acid metabolism^3,4^. While SCFAs and amino acid metabolism contribute to gut homeostasis, the role of microbially derived long-chain fatty acids (LCFAs) in promoting gut mucosal recovery remains largely unexplored.

The gut-associated lymphoid tissue (GALT) is an early target of human immunodeficiency virus (HIV) infection, resulting in rapid depletion of gut mucosal CD4^+^ T cells and epithelial barrier disruption^5^. Establishment of viral reservoirs and mucosal inflammation occurs early in both HIV and simian immunodeficiency virus (SIV) infections^6,7^. Gut epithelial disruption impairs epithelial–immune cell interactions thereby impeding mucosal immunity^8^. Combination anti-retroviral therapy (ART) effectively suppresses viral replication and reduces mortality in HIV-infected individuals but neither eradicates viral reservoirs nor can it fully restore host gut mucosal structure and immunity^9–12^. HIV and SIV infections are associated with alterations to gut microbial communities, including loss of beneficial microbial taxa, leading to increased susceptibility to opportunistic infections and accelerating disease progression^13–15^. We previously demonstrated that intraluminal administration of *Lactiplantibacillus plantarum* into the SIV-inflamed gut restored epithelial barrier integrity and reduced mucosal inflammation^4,9^. However, clinical trials of probiotic supplementation in HIV-infected individuals have yielded inconsistent outcomes, suggesting that specific bioactive microbial metabolites may exert more consistent therapeutic effects^16,17^. We sought to identify bioactive microbially derived metabolites that induce gut epithelial repair thereby supporting the mucosal immune system to corral and silence the virus. We hypothesized that gut mucosal restoration would increase the efficacy of ART.

In this study, we discovered the microbially derived octadecanoid-hydroxy-fatty-acid metabolite, 10-hydroxystearic acid (10-HSA), as a key mediator of gut epithelial barrier repair. It was most predominantly produced in the SIV-inflamed gut microenvironment immediately following *L. plantarum* administration and was linked to gut epithelial renewal. Our data show that 10-HSA directly binds to and activates peroxisome proliferator-activated receptor-α (PPARα), promoting mitochondrial bioenergetics, host lipid metabolism and histone crotonylation associated with gut epithelial regeneration. Combination of 10-HSA treatment with ART markedly accelerated viral suppression and gut epithelial barrier renewal. These findings identify a previously unrecognized role for microbially derived 10-HSA in the repair of virally inflamed gut and define an accelerated combination therapy (ACT) strategy that integrates anti-retroviral control with microbial-metabolite-driven gut mucosal recovery for durable viral silencing.

## Results

### Microbially derived 10-HSA binds PPARα and restores gut epithelium

**Fig. 1:**
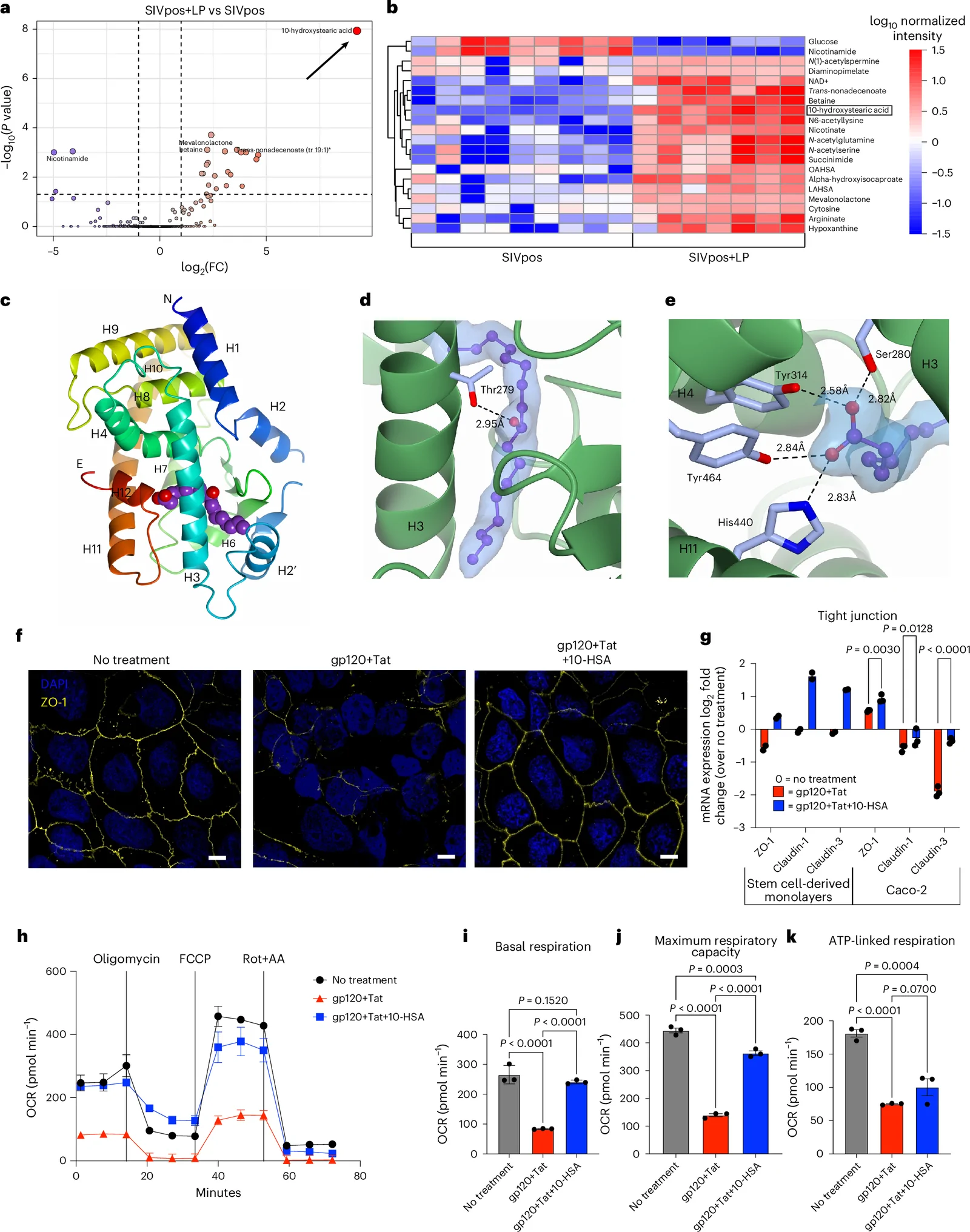
Repair of the gut epithelial barrier through PPARα activation by microbially derived bioactive octadecanoid lipid. **a**, Volcano plot of metabolites between SIVposLP (LP, *L. plantarum*; *n* = 7 biological replicates) and SIVpos (*n* = 9 biological replicates) groups. Data analysed using Student’s *t*-test. **b**, Heat map showing the top 20 significantly produced metabolites between SIVposLP (*n* = 7 biological replicates) and SIVpos (*n* = 9 biological replicates) groups. **c**, X-ray crystallography data showing 10-HSA (purple) binding PPARα (green) with hydrogen bonds shown in yellow. **d**, Closeup of the 10-OH forming a hydrogen bond with the T279 amino acid residue in PPARα. **e**, Closeup of the carboxylic acid end of 10-HSA forming hydrogen bonds with H440, Y464, Y314 and S280 amino acid residues in the PPARα protein. Numbers next to hydrogen bonds indicate distance of bond in Å. **f**, ZO-1 (yellow) immunofluorescence staining in Caco-2 cells following 6 h of treatment with 10-HSA and HIV viral antigens (*n* = 3 per group). **g**, Tight junction gene expression in SCDM ex vivo (*n* = 2 biological replicates) and Caco-2 (*n* = 3 biological replicates) in vitro following 6 h of treatment with 10-HSA and HIV viral antigens. Two-way ANOVA was performed. **h**–**k**, Seahorse OCR assay output in Caco-2 (*n* = 3 biological replicates) following 6 h of treatment with 10-HSA and HIV viral antigens. Data analysed using one-way ANOVA with *n* = 3 biological replicates. Scale bar, 5 μm. All data are presented as mean ± s.e.m. Source data

**Extended Data Fig. 1:**
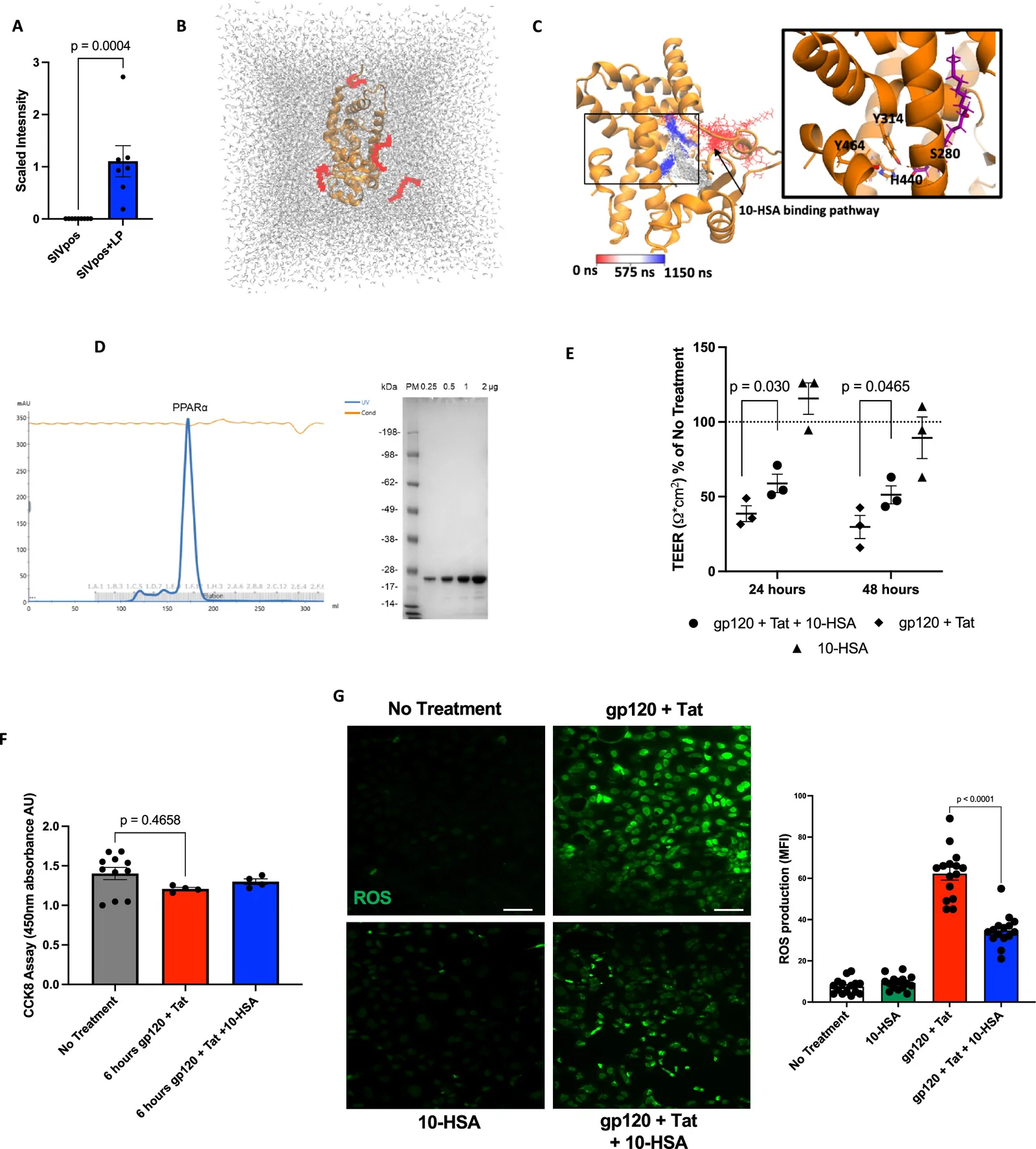
Activation of PPARα linked to gut barrier tight junction function. (**A**) Luminal contents of 10-HSA in LP treated vs untreated loops (n = 7 biological replicates SIVpos+LP, n = 10 biological replicates SIVpos), data analyzed by unpaired one-way t test. (**B**) Setup for GAMD simulation, PPARα shown in orange, four 10-HSA molecules shown in red, water shown in grey. (**C**) Magnified view of the PPARα binding pocket showing 10-HSA (purple) engaged with canonical agonist-activating residues. The carboxylic acid moiety of 10-HSA forms interactions with Y314, Y464, H440, and S280 within the LBD, consistent with the hydrogen bonding network identified by X-ray crystallography of the 10-HSA–PPARα complex. **D**) Chromatogram (left) and SDS-PAGE (right) analysis of untagged PPARα-LBD protein following polishing by Size-Exclusion Chromatography. To ensure specific purification, samples were run on SDS-PAGE with 4 masses (0.25, 0.5, 1, and 2 μg). (**E**) TEER results after 24 and 48 hours of 10-HSA treatment with HIV viral proteins (n = 3 biological replicates per group per timepoint). Data analyzed by unpaired one-way t test. (**F**) CCK8 cell viability assay for Caco2 cells treated with gp120+Tat and 10-HSA (n = 11 biological replicates No treatment, n = 4 biological replicates gp120 + Tat treatment, n = 4 biological replicates gp120+Tat+10-HSA treatment). Data analyzed by unpaired one-way t test. (**G**) Radical oxygen species (ROS) levels as determined by CellROX green assay done on Caco2 cells with HIV viral proteins and 10-HSA with semi-quantification of mean fluorescence intensity (n = 15 cells quantified per treatment). Experiment was repeated. Scale bar = 10 micron. Data analyzed by unpaired one-way t test. Data are presented as mean values +/- SEM. Source data

Administration of *L. plantarum* directly into intestinal loops of SIV-infected macaques rapidly restored gut epithelial barriers through activation of PPARα^9^. To identify *L. plantarum*-derived metabolites associated with this reparative effect, we performed untargeted metabolomics of gut luminal contents and evaluated *L. plantarum* metabolic activity induced in response to the inflamed gut. The data showed 10-HSA as the most upregulated metabolite following *L. plantarum* treatment (Fig. 1a,b, Supplementary Table 1 and Extended Data Fig. 1a). Gaussian accelerated molecular dynamics (GaMD) simulations showed that 10-HSA inserts into the PPARα ligand binding domain (LBD) and stabilizes through binding H440, Y464, Y314 and S280 residues (Extended Data Fig. 1b,c). To confirm the direct 10-HSA–PPARα binding interaction, we performed X-ray crystallography of the 10-HSA–PPARα complex with a 2.1 Å resolution (Supplementary Table 2 and Extended Data Fig. 1d). Structural analysis revealed stable binding of 10-HSA through hydrogen-bond formation in the LBD of PPARα (Fig. 1c). Specifically, the 10-hydroxyl group in 10-HSA forms a hydrogen bond with T279 in the PPARα protein (Fig. 1d). The 10-HSA carboxylic acid group forms bonds with the PPARα H440, Y464, Y314 and S280 amino acid residues (Fig. 1c–e). These five amino acid residues contribute exclusively to agonist activation of PPARα^18^. Our data demonstrate that GaMD can independently replicate X-ray crystallography protein–ligand interactions.

We assessed the bioactivity of the microbially derived 10-HSA (500 μM) in human gut epithelial models, Caco-2 cells in vitro and duodenal stem cell-derived epithelial monolayers (SCDM) ex vivo following exposure to HIV antigens (Envelope protein gp120 and Tat)^9^. Exposure to HIV viral antigens caused substantial disruption of the ZO-1 tight junction structure in Caco-2 cells (Fig. 1f)^9^. In contrast, co-treatment with 10-HSA preserved epithelial junction structural integrity (Fig. 1f). Trans-epithelial electrical resistance (TEER) measurements showed significant recovery of epithelial barrier function with 10-HSA treatment (Extended Data Fig. 1e). Transcriptional analysis of SCDM and Caco-2 cells revealed that viral antigens caused the loss of gene expression regulating tight junction proteins, while 10-HSA treatment restored their expression (Fig. 1g). In parallel, viral antigen exposure for 6 h led to significant reduction in basal and maximum oxygen respiration activity of Caco-2 cells but did not alter cell viability (Fig. 1h–j and Extended Data Fig. 1f)^9^. Treatment with 10-HSA resulted in significantly increased basal and maximum respiration as well as ATP synthase-linked respiration in viral antigen-exposed cells (Fig. 1h–k). These data reflecting increased mitochondrial function were accompanied by reduced reactive oxygen species (ROS) levels (Extended Data Fig. 1g). Our findings demonstrate that 10-HSA binds to PPARα, promotes mitochondrial function and restores gut epithelial barrier structure during viral disruption.

### Activation of PPARα protects against HIV antigen-induced epithelial damage

**Extended Data Fig. 2:**
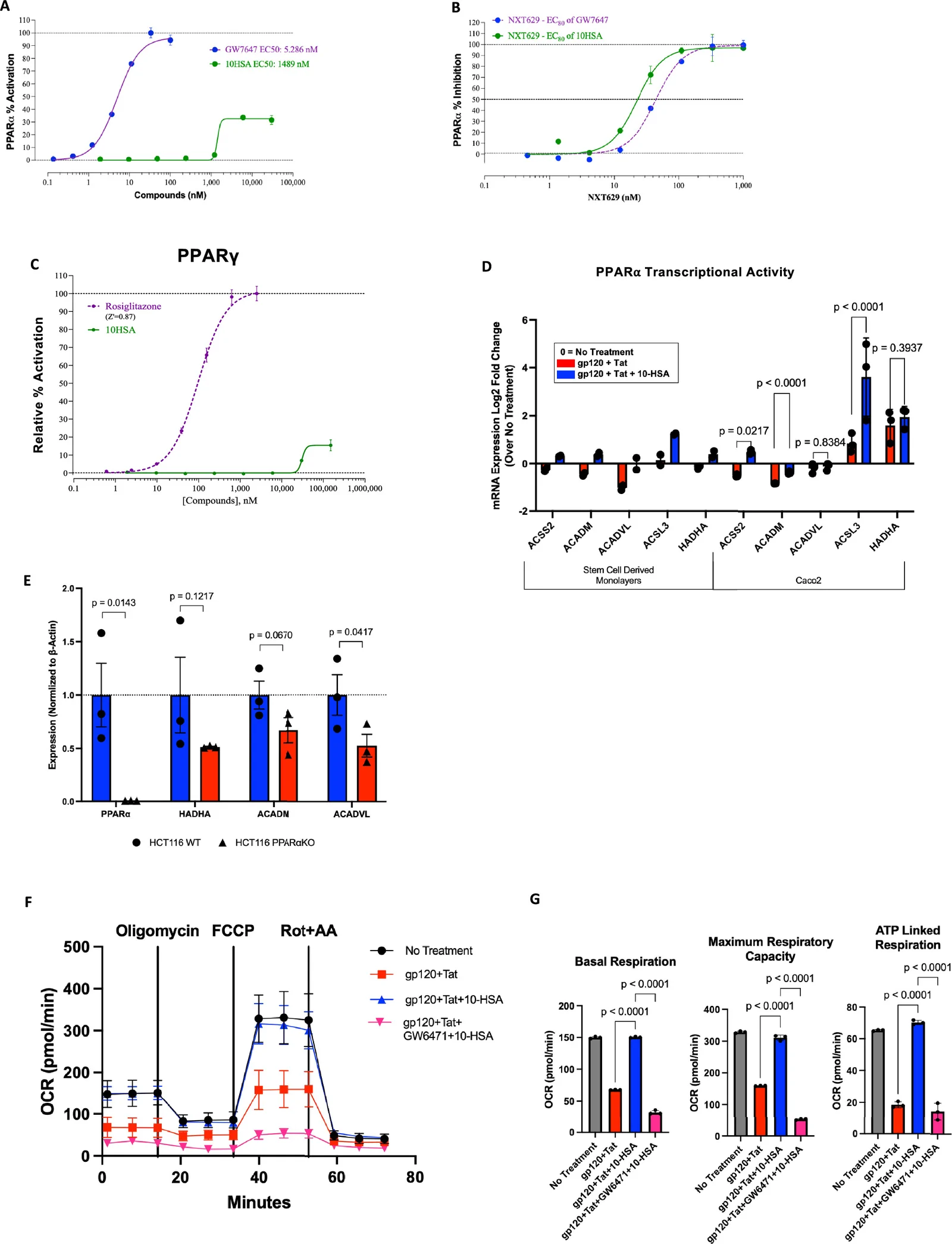
PPARα is required for mediating 10-HSA effects. (**A**) Luciferase reporter assay showing PPARα activation by 10-HSA (n = 3 biological replicates per concentration). (**B**) Luciferase reporter assay showing suppression of PPARα by NXT629 inhibitor (n = 3 biological replicates per concentration). (**C**) Luciferase reporter gene assay showing PPARγ activation from Rosiglitazone and 10-HSA (n = 3 biological replicates per concentration). (**D**) RT-qPCR data showing PPARα transcriptional activity after 10-HSA treatment in Caco2 (n = 3 biological replicates) and SCDM cells (n = 2 biological replicates) with HIV viral proteins. Data analyzed with two-way ANOVA. (**E**) HCT-116 cell RT-qPCR showing expression levels of known PPARα regulated genes with and without PPARα knockout (n = 3 biological replicates per group per cell type). Data analyzed with two-way ANOVA. (**F**) Seahorse OCR assay on Caco2 cells (n = 3 biological replicates per group) with HIV viral proteins, 10-HSA, and known PPARα antagonist GW6471. (**G**) Analysis of Seahorse OCR assay (n = 3 biological replicates per group). Data analyzed with one-way ANOVA. Data are presented as mean values +/- SEM. Source data

To assess PPARα activation by the microbially derived 10-HSA, we used a reporter cell line expressing a PPARα-driven luciferase reporter. A dose-dependent increase in PPARα transcriptional activity was detected in 10-HSA-treated luciferase reporter cells (Extended Data Fig. 2a). This activation was fully abrogated by the PPARα-specific pharmacological inhibitor NXT629 (Extended Data Fig. 2b). We next examined whether 10-HSA activates other PPAR isoforms. 10-HSA also activated PPARγ; however, it required substantially higher concentrations than those required for PPARα activation, establishing PPARα as the primary mediator for 10-HSA-driven gut mucosal repair (Extended Data Fig. 2a,c).

**Fig. 2:**
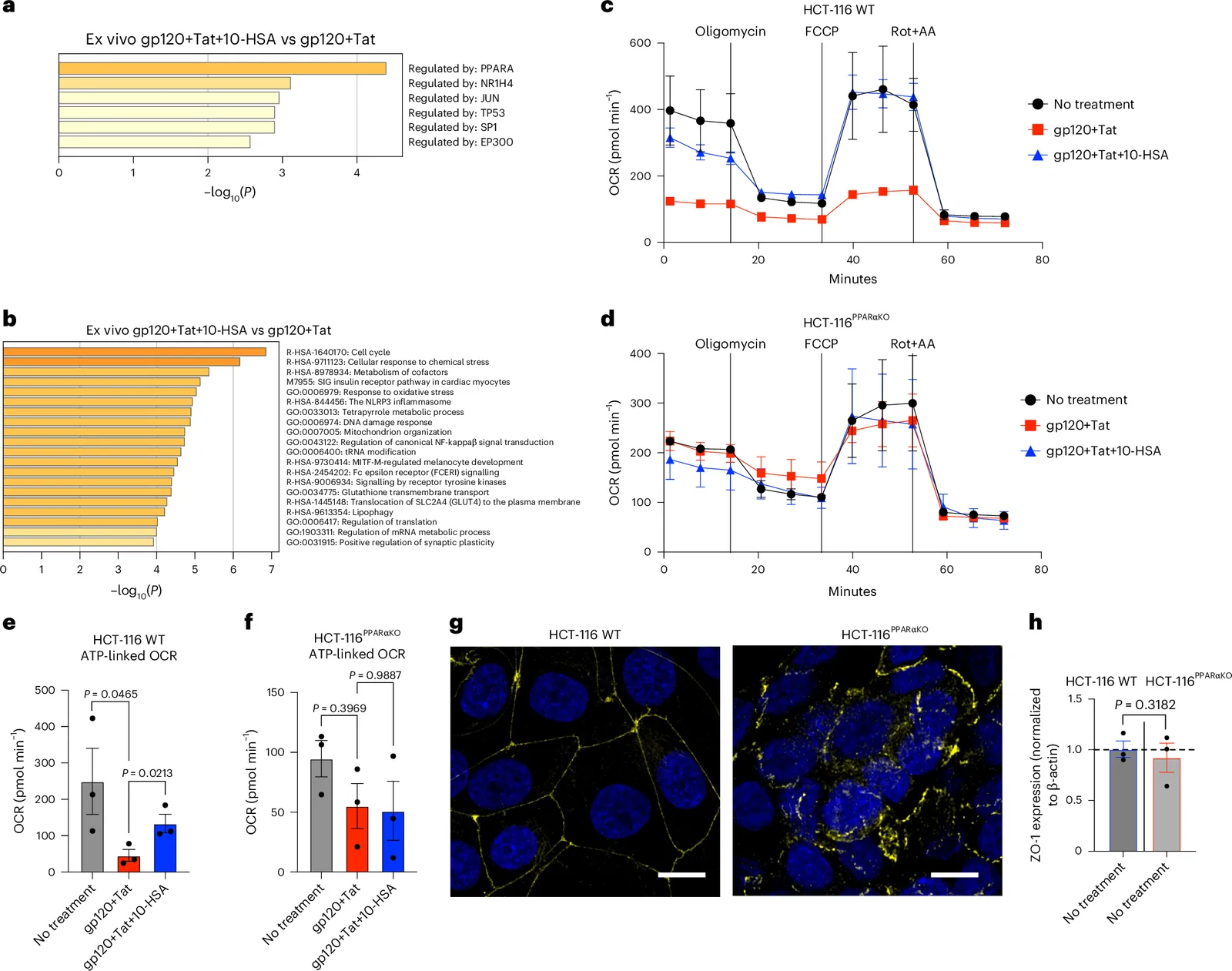
Gut epithelial barrier repair is mediated through PPARα-driven mitochondrial function. **a**, TRRUST analysis of upregulated genes from RNA sequencing in SCDM following 10-HSA and HIV antigen treatments. **b**, Pathway analysis of upregulated genes in gp120 + Tat + 10-HSA-treated SCDM compared to gp120 + Tat cells. **c**,**d**, Seahorse OCR assay output of HCT-116 (**c**) and HCT-116^PPARαKO^ (**d**) cells following 30 min of HIV viral protein exposure with 10-HSA (*n* = 3 biological replicates per group per cell type). **e**,**f**, ATP synthase-linked OCR in HCT-116 (**e**) and HCT-116^PPARαKO^ (**f**) cells with HIV viral protein treatment and 10-HSA. Seahorse assay was conducted with *n* = 3 biological replicates for each treatment group, and results were analysed using one-way unpaired *t*-test. **g**, Immunofluorescence of ZO-1 in HCT-116 and HCT-116^PPARαKO^ cells (ZO-1 in yellow, *n* = 3 biological replicates per cell type). **h**, mRNA expression of ZO-1 in HCT-116 and HCT-116^PPARαKO^ cells as determined by qRT–PCR (*n* = 3 biological replicates per cell type). Data analysed using one-way unpaired *t*-test. Scale bar, 5 μm. Data are presented as mean ± s.e.m. Source data

To identify downstream transcriptional effects on mucosal gene networks, we performed RNA-seq analysis on HIV antigen-exposed SCDM treated with 10-HSA. Transcription Regulatory Relationships Unravelled by Sentence-based Text mining (TRRUST) analysis identified enrichment for PPARα regulated gene expression (*q* = 0.0316) in HIV antigen-exposed cells treated with 10-HSA as compared to only viral antigen-exposed cells (Fig. 2a). Pathway analysis of upregulated genes in SCDM following 10-HSA treatment revealed enrichment for genes associated with mitochondrial organization (*q* = 0.0366) and response to oxidative stress (*q* = 0.0266), as well as lipophagy-associated pathways (*q* = 0.054, Fig. 2b)^19^. The increase in PPARα-regulated gene expression was detected in both SCDM and Caco-2 cells by quantitative PCR with reverse transcription (RT-qPCR) (Extended Data Fig. 2d).

To directly establish the role of PPARα in HIV antigen-mediated pathogenic effects, we utilized a human gut epithelial PPARα-knockout cell line, HCT-116^PPARαKO^. Loss of PPARα expression and its downstream transcriptional targets in these cells validated the functional PPARα knockout (Extended Data Fig. 2e). In wild-type (WT) HCT-116 cells, HIV antigen exposure significantly reduced basal, maximal and ATP synthase-linked respiration as measured by oxygen consumption rate (OCR) assay, while all three parameters were restored with 10-HSA treatment (Fig. 2c,e). In contrast, PPARα^KO^ cells did not exhibit significant reductions in basal, maximal or ATP synthase-linked cellular respiration following HIV antigen exposure, and 10-HSA treatment did not alter these parameters (Fig. 2d,f). In PPARα^KO^ cells, viral antigens marginally reduced ATP synthase-linked OCR, while the 10-HSA treatment had minimal impact (Fig. 2f). Consistent with these findings, pharmacological inhibition of PPARα with GW6471 in Caco-2 cells abolished the ability of 10-HSA to restore mitochondrial respiration in HIV antigen-exposed cells (Extended Data Fig. 2f,g). In WT HCT-116 monolayers, ZO-1 protein displayed continuous localization along the epithelial cell borders (Fig. 2g). PPARα^KO^ cells showed discontinuous and disorganized ZO-1 (Fig. 2g). Moreover, PPARα^KO^ cells did not show detectable change in ZO-1 expression compared to WT HTC-116 cells (Fig. 2h). These findings showed that PPARα deficiency leads to inefficient ZO-1 transport and impairs its localization and structural organization. Our data demonstrate that HIV antigens impair mitochondrial respiration and epithelial integrity through disruption of PPARα signalling which was restored with 10-HSA treatment.

### Metabolic and molecular signatures of gut epithelial barrier repair in SIV-infected macaques

**Fig. 3:**
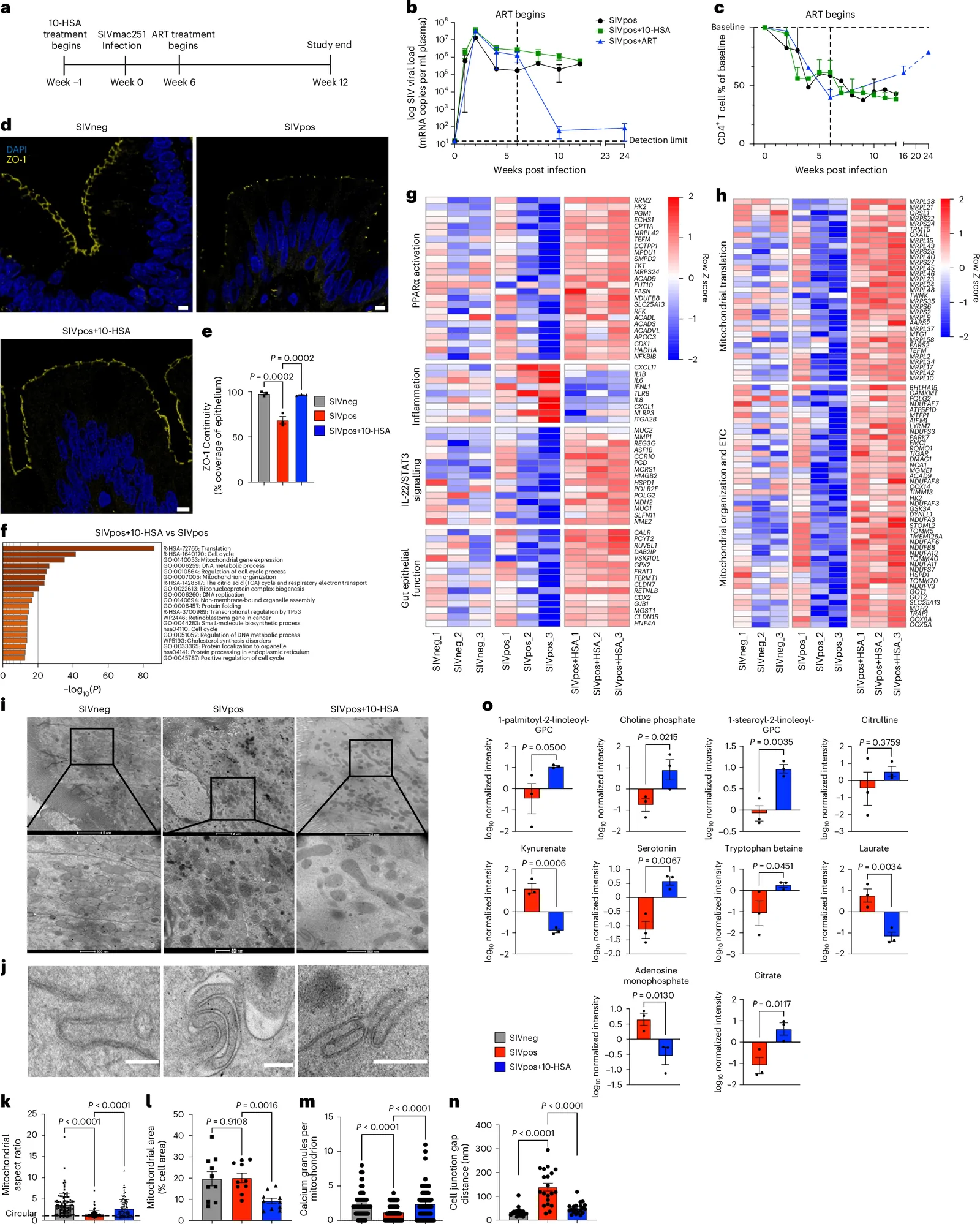
In vivo restoration of gut epithelial barrier and mitochondrial bioenergetics in SIV infection following 10-HSA treatment. **a**, Macaque study design and treatments, created with Biorender. **b**, Longitudinal viral load data from peripheral blood (*n* = 3 biological replicates per group). **c**, Longitudinal CD4^+^ T-cell percent change from CBC data in peripheral blood (*n* = 3 biological replicates per group). **d**, Representative images of immunofluorescence for ZO-1 (yellow) in small intestine of SIVneg (*n* = 3), SIVpos (*n* = 3) and 10-HSA (*n* = 3) treated animals at the study end timepoint (*n* = 3 biological replicates per group). Scale bar, 5 μm. **e**, ZO-1 continuity across the gut epithelium (*n* = 3 biological replicates per group). Data analysed using one-way ANOVA (*n* = 3 biological replicates per group). **f**, Pathway analysis of significantly increased genes from SIVpos-HSA vs SIVpos groups as detected by RNA-seq data (*n* = 3 biological replicates per group). **g**, Altered gene expression in 10-HSA-treated SIVpos animals compared to SIVpos animals and SIVneg animals. **h**, Altered genes in jejunal mucosal tissue of 10-HSA-treated SIVpos animals compared to SIVpos animals alone pertaining to mitochondrial related genes. **i**, Representative images of mitochondria in apical enterocytes as viewed by TEM of intestinal tissue at the study endpoint (*n* = 3 biological replicates per group). **j**, Representative images of cellular gap junctions in apical enterocytes from jejunum tissue at the study endpoint (scale bar, 250 nm; *n* = 3 biological replicates per group). **k**, Mitochondrial aspect ratio across treatments (*n* = 100 mitochondria per treatment group) quantified in ImageJ. **l**, Mitochondrial area per cell across treatments quantified in ImageJ (*n* = 10 cells per treatment, *n* = 3 biological replicates per group). **m**, Dense body granule count across treatments quantified in ImageJ (*n* = 75 mitochondria per treatment, *n* = 3 biological replicates per group). **n**, Cell gap junction width across treatments quantified in ImageJ (*n* = 20 cell–cell gaps per treatment group, *n* = 3 biological replicates per group). For all mitochondrial assessments, data were analysed using one-way ANOVA. **o**, Metabolites from untargeted metabolomics associated with gut and mitochondrial function (*n* = 3 biological replicates per group), data analysed using unpaired one-way *t*-test. Data are presented as mean ± s.e.m. Source data

To determine the impact of the microbially derived 10-HSA treatment on gut mucosal repair in vivo, we evaluated SIV-infected non-human primates (NHP) following 10-HSA treatment for 13 weeks (SIVpos-HSA, *n* = 3) and compared them with untreated SIV-infected controls (SIVpos, *n* = 3), SIV-infected animals receiving ART (SIVpos + ART, *n* = 3) and SIV-negative controls (SIVneg, *n* = 3; Fig. 3a). Plasma viral loads were comparable between SIVpos and SIVpos-HSA animals (Fig. 3b). Peripheral CD4^+^ T-cell numbers decreased in SIVpos-HSA animals, whereas ART suppressed viral loads and partially restored CD4^+^ T-cell numbers (Fig. 3b,c). No significant differences were found in gut mucosal CD4^+^ T-cell populations between SIVpos animals in the presence or absence of 10-HSA treatment (Supplementary Fig. 1a).

**Extended Data Fig. 3:**
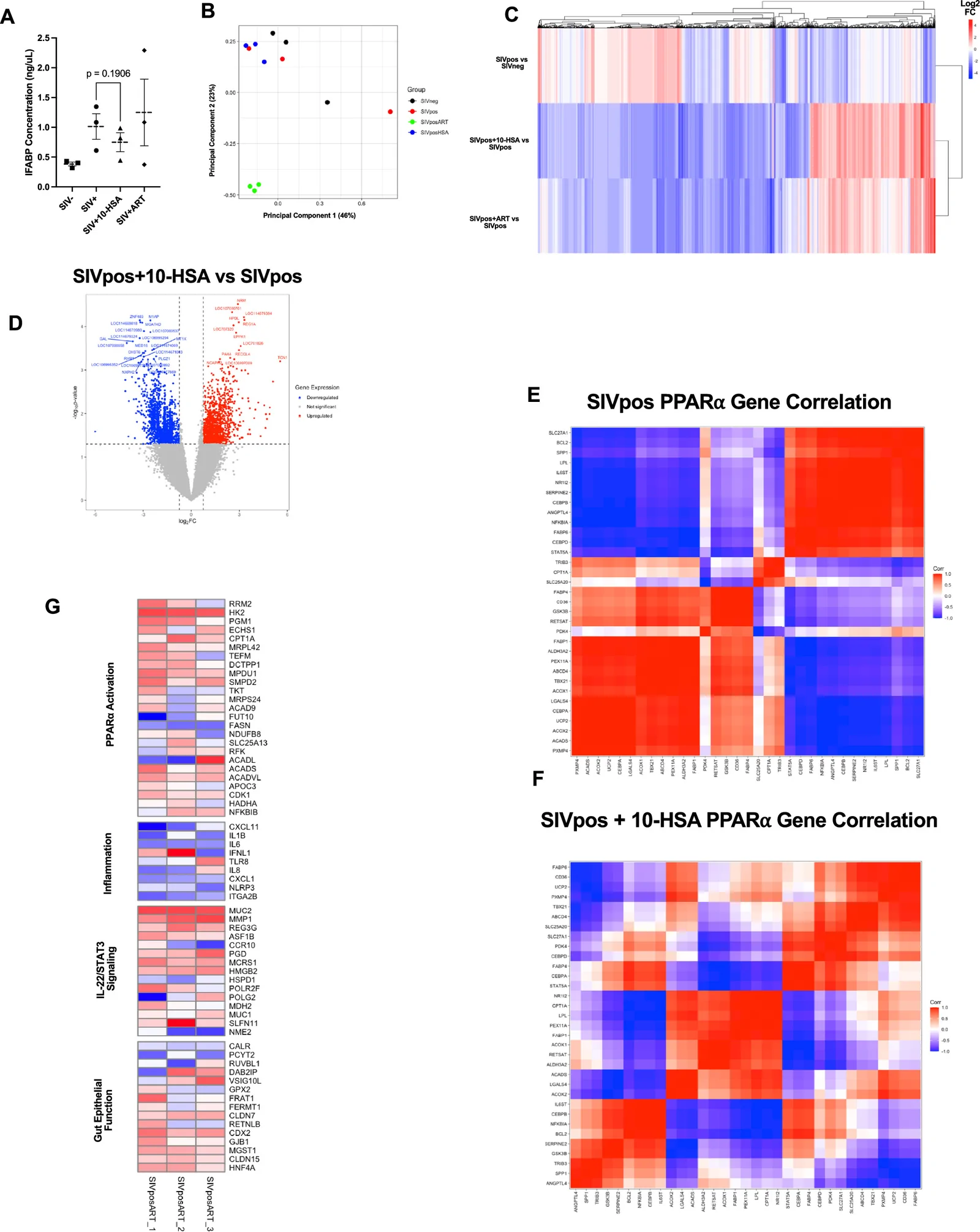
Antiretroviral Therapy does not fully recover gut mitochondrial related gene expression in SIV infection. (**A**) IFABP concentration in peripheral blood serum of animals in the study (n = 3 biological replicates for SIVneg, SIVpos, SIVpos+ART, and SIVpos+10-HSA). Data analyzed by unpaired one-way t test. (**B**) PCA plot of RNAseq data. (**C**) Heatmap of all changes to gene expression by comparison (n = 3 biological replicates for SIVneg, SIVpos, SIVpos+ART, and SIVpos+10-HSA). (**D**) Volcano plot of differentially expressed genes in SIVpos+10-HSA vs SIVpos animals (n = 3 biological replicates per group). (**E**) Gene correlation pattern of known PPARα regulated genes in SIVpos animals (n = 3 biological replicates). (**F**) Gene correlation pattern of known PPARα regulated genes in SIVpos+10-HSA animals (n = 3 biological replicates). (**G**) ART treated animals’ gene expression of relevant pathways (n = 3 biological replicates). Data are presented as mean values +/- SEM. Source data

Assessment of the gut epithelial barrier structure revealed intact and continuous ZO-1 lattice structure in SIVneg animals, while SIVpos animals displayed pronounced ZO-1 disorganization and fragmentation (Fig. 3d,e). In contrast, 10-HSA treatment prevented the disruption of the ZO-1 epithelial structure (Fig. 3d,e). Structural organization of the tight junction protein Claudin-3 was also enhanced in the gut epithelium of SIVpos-HSA animals compared to SIVpos animals (Supplementary Fig. 1c,d). We previously reported that gut epithelial barrier recovery in HIV and SIV infections during ART is not consistent or complete^9^. Therefore, we measured circulating levels of intestinal fatty acid-binding protein (IFABP), a marker of epithelial damage and intestinal permeability. We found that IFABP levels were reduced in SIVpos animals following 10-HSA treatment compared to untreated SIVpos animals (Extended Data Fig. 3a).

To characterize transcriptional changes associated with 10-HSA treatment, we performed RNA-seq on gut mucosal tissues. Principal component analysis (PCA) showed separate clustering of SIVpos-HSA animals from SIVpos controls (Extended Data Fig. 3b–d). Differential expression analysis identified a significant upregulation of 1,190 genes (false discovery rate (FDR) < 0.15, *P* < 0.05) in SIVpos-HSA animals compared to SIVpos controls. Pathway analysis of these genes revealed enrichment of mitochondrial translation and organization as well as DNA metabolism and cell cycle activation in 10-HSA-treated animals (Fig. 3f)^20^. Genes associated with lipid metabolism and PPARα signalling were also upregulated in SIVpos-HSA animals (Fig. 3g). PPARα signalling suppresses NF-κB activation through increased expression of IκB^21^. Increased expression of *NFKBIB* transcripts, encoding the beta subunit of IκB, and decreased expression of pro-inflammatory mediators were detected in SIVpos-HSA animals compared to SIVpos animals (Fig. 3g). Pearson’s correlation analysis of PPARα-regulated genes revealed distinct clustering patterns between SIVpos and SIVpos-HSA groups (Extended Data Fig. 3e,f). In SIVpos-HSA animals, strong positive correlations were detected for the gene pairs including *FABP1* and *FABP6*, *FABP4* and *NFKBIA*, and *CPT1A* and *RETSTAT*, while SIVpos animals showed negative correlation between the same genes (Extended Data Fig. 3e,f).

Analysis of gut epithelium-associated gene expression showed upregulation of critical tight junction and innate immune defence genes *CLDN7, CLDN15, MUC2* and *RETNLB* in SIVpos-HSA animals compared to SIVpos controls (Fig. 3g)^9^. Increased activity of IL-22-driven STAT3 signalling was detected in 10-HSA-treated animals (Fig. 3g). IL-22-induced gene expression is vital for preserving epithelial integrity and is suppressed in SIV infection (Fig. 3g). *CCR10* expression was elevated in SIVpos-HSA compared to SIVpos animals (Fig. 3g). Since CCR10 is expressed on Th22 cells and mediates chemotaxis, these data indicated an enhanced mucosal Th22 response following 10-HSA treatment^22^. ART-treated animals displayed only partial restoration of the critical transcriptomic pathways impaired by SIV infection (Extended Data Fig. 3g).

### Restoration of mitochondrial bioenergetics and gut epithelial integrity in vivo

**Extended Data Fig. 4:**
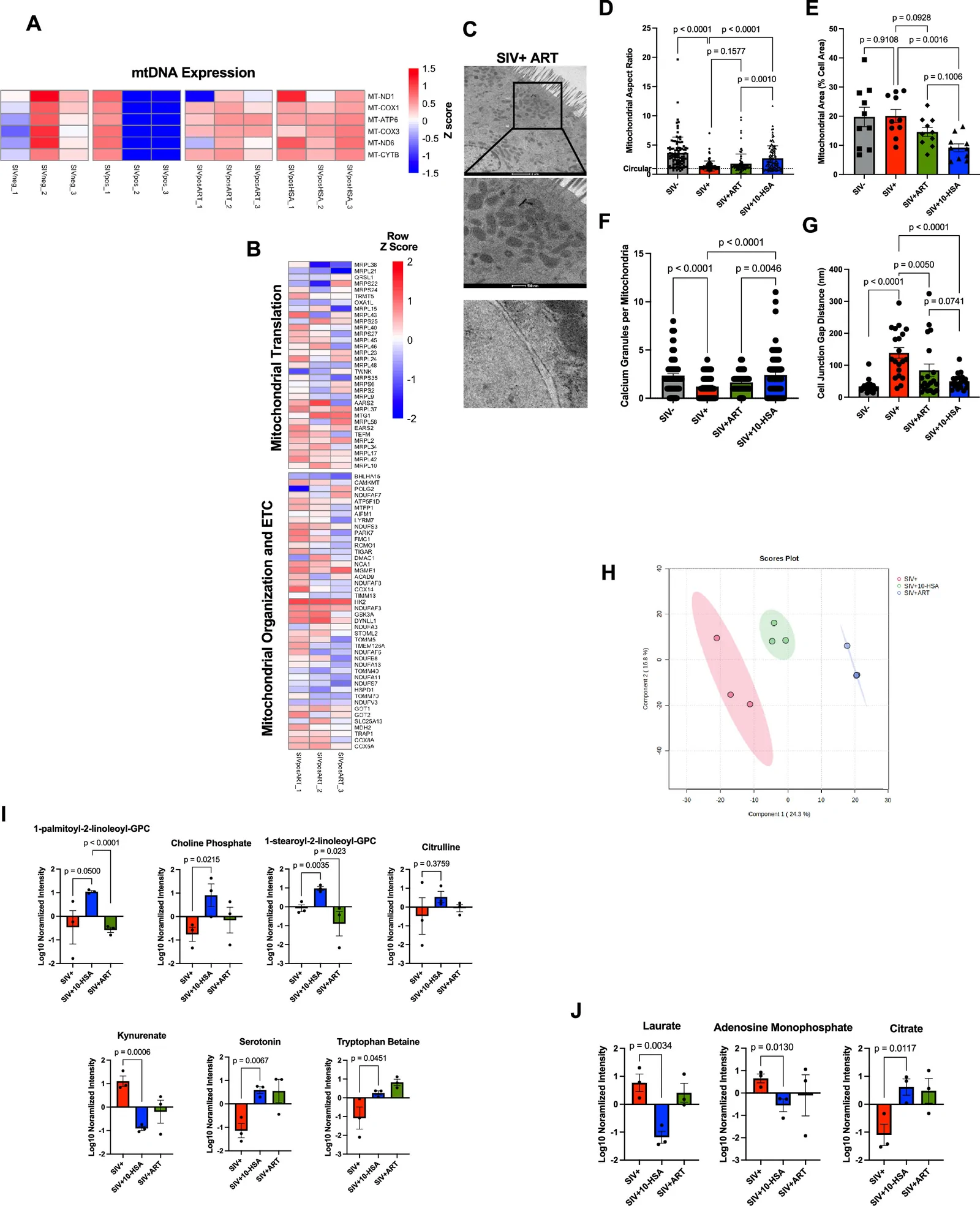
ART does not fully recover gut epithelial mitochondrial morphology in SIV infection. (**A**) Z-score plot showing mitochondrial gene expression (n = 3 biological replicates per group) and (**B**) ART (n = 3 biological replicates) treated animals’ translation and organization. (**C**) Transmission electron micrographs of mitochondria in gut enterocytes from ART (n = 3 biological replicates) treated animals. Data on (**D**) mitochondrial aspect ratio, (**E**) mitochondria area/cell, (**F**) calcium granules, (**G**) cell junction width from the main text Figures with the addition of ART treated (n = 3 biological replicates) animals. (**H**) PLS-DA plot of metabolomic data (n = 3 biological replicates per group). Levels of known important (**I**) gut metabolites and (**J**) mitochondrially related metabolites from main text Figures with the inclusion of ART treated animals (n = 3 biological replicates per group). For all mitochondrial assessments data was analyzed by one-way ANOVA. Metabolomic data analyzed by unpaired one-way t-test. Data are presented as mean values +/- SEM. Source data

To determine whether mitochondrial integrity and functionality in the SIV-inflamed gut were restored by the microbially derived 10-HSA treatment, we integrated transcriptomics, metabolomics and ultrastructural assessments. Transcriptomic analysis revealed upregulation of mitochondrial regeneration and function-associated genes in SIVpos-HSA animals compared to SIVpos controls (Fig. 3h). Expression of mitochondrial translation pathways was highly upregulated in SIVpos-HSA animals, along with enrichment of gene expression regulating the electron transport chain (ETC), mitochondrial organization and protein import (Fig. 3h). Consistent with these findings, increased expression of six mitochondrially encoded genes associated with ETC and ATP synthesis was observed (Extended Data Fig. 4a).

We utilized transmission electron microscopy (TEM) to visualize mitochondrial morphology in gut epithelial cells. Consistent with our previous findings, untreated SIV-infected animals displayed more circular mitochondria compared with SIVneg controls (Fig. 3i,k)^9^. In contrast, 10-HSA treatment restored normal tubular mitochondrial morphology while reducing overall mitochondrial area in the cell (Fig. 3i,k,l). Mitochondria of SIVpos-HSA animals showed increased calcium granule density compared to SIVpos animals (Fig. 3m)^9^. TEM analysis confirmed that 10-HSA treatment led to restoration of the gut epithelial cell–cell junctions compared to SIVpos animals and correlated with mitochondrial recovery (Fig. 3j,n). In contrast, animals receiving ART only showed modest to minimal recovery of mitochondria-associated gene expression, mitochondrial morphology, and epithelial cell–cell junctions and integrity (Extended Data Fig. 4b–g).

Untargeted serum metabolomic profiling distinguished experimental groups (Extended Data Fig. 4h). Citrulline biosynthesis occurs primarily in gut enterocytes and is a robust measurement of gut epithelial function^23^. Metabolites associated with gut epithelial function including citrulline, choline phosphate and specific phosphatidylcholines, were elevated in SIVpos-HSA compared to SIVpos controls (Fig. 3o). Serotonin levels increased in serum of 10-HSA-treated animals (Fig. 3o). Serum serotonin levels were also elevated following 10-HSA treatment, indicating enhanced enteroendocrine cell activity and function in the gut^24^. In addition, 10-HSA treatment also increased tryptophan betaine while decreasing kynurenate levels, indicating remodelling of tryptophan metabolism diverted from the immunosuppressive kynurenine pathway (Fig. 3o)^4^. Levels of mitochondrially linked energy metabolites laurate and adenosine monophosphate (AMP) accumulated in SIVpos animals, indicating mitochondrial dysfunction and impaired fatty acid oxidation, while their levels were significantly reduced in SIVpos-HSA animals (Fig. 3o)^25^. Further, circulating citrate levels were increased following 10-HSA treatment, suggesting enhanced tricarboxylic acid (TCA) cycle activity (Fig. 3o)^26^. ART alone resulted in only partial restoration of gut-associated and mitochondria-associated metabolic profiles (Extended Data Fig. 4i,j).

### ACT accelerates viral suppression and gut mucosal restoration

**Fig. 4:**
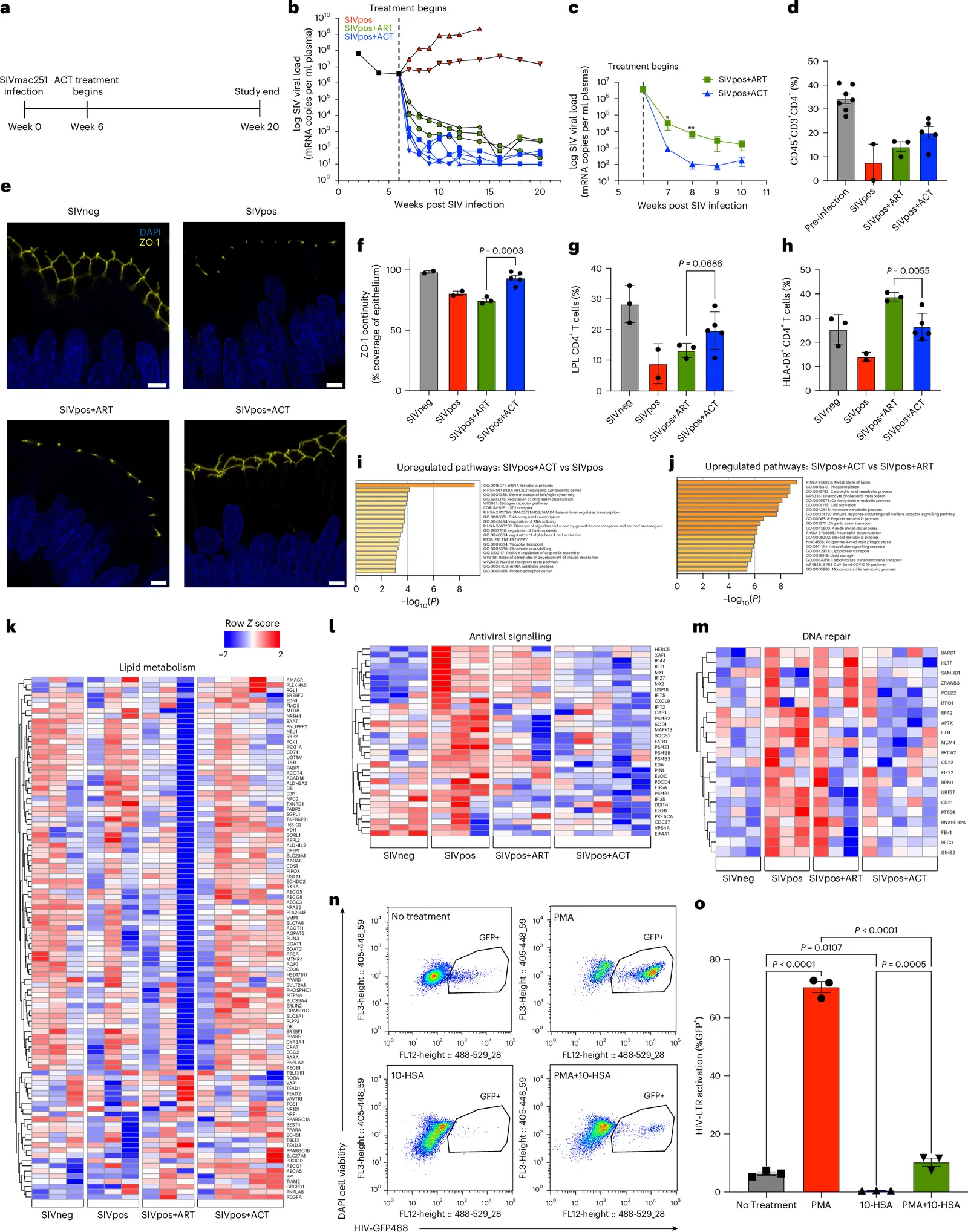
Accelerated viral suppression by combining ART with 10-HSA treatment in SIV infection in vivo. **a**, Animal study design, created with Biorender. **b**, Viral load data from peripheral blood plasma shown longitudinally over the course of the study (*n* = 2 (SIVpos), *n* = 3 (SIVpos + ART), *n* = 5 (SIVpos + ACT) biological replicates). **c**, Viral load data from the first 4 weeks of treatments (*P = 0.0365, **P = 0.0098; *n* = 3 (SIVpos + ART), *n* = 5 (SIVpos + ACT) biological replicates; one-way *t*-test). **d**, CD4^+^ T-cell percentages from colo-rectal biopsies taken at 2 weeks post treatment (8 weeks post infection; *n* = 7 (pre-infection timepoint), *n* = 3 (SIVpos + ART), *n* = 5 (SIVpos + ACT), *n* = 2 (SIVpos) biological replicates). **e**, Representative images of jejunum tissue showing immunofluorescence of ZO-1 (yellow) stain. Scale bar, 5 μm. **f**, ZO-1 continuity across the gut epithelium analysed using one-way *t*-test (*n* = 2 (SIVneg), *n* = 2 (SIVpos), *n* = 3 (SIVpos + ART), *n* = 5 (SIVpos + ACT) biological replicates). **g**, CD4^+^ T-cell percentages in isolated jejunum LPLs analysed using one-way *t*-test (*n* = 3 (SIVneg), *n* = 2 (SIVpos), *n* = 3 (SIVpos + ART), *n* = 5 (SIVpos + ACT) biological replicates). **h**, HLA-DR^+^ CD4^+^ T cells in isolated jejunum LPLs analysed using one-way *t*-test (*n* = 3 (SIVneg), *n* = 2 (SIVpos), *n* = 3 (SIVpos + ART), *n* = 5 (SIVpos + ACT) biological replicates). **i**, Upregulated gut mucosal transcriptomic pathways in SIVpos + ACT (*n* = 5 biological replicates) vs SIVpos (*n* = 3 biological replicates) animals. **j**, Upregulated gut mucosal transcriptomic pathways in SIVpos + ACT (*n* = 5 biological replicates) vs SIVpos + ART (*n* = 3 biological replicates). **k**–**m**, Heat maps illustrating lipid metabolism (**k**), antiviral interferon signalling (**l**) and DNA repair pathways (**m**) from RNA-seq data. **n**, Representative flow cytometry gating for GFP + J-Lat cells indicating HIV-LTR reactivation. **o**, Quantification of GFP + J-Lat cells from experiment performed in triplicate (*n* = 3 biological replicates per group), with data analysed using one-way ANOVA. Data are presented as mean ± s.e.m. Source data

**Extended Data Fig. 5:**
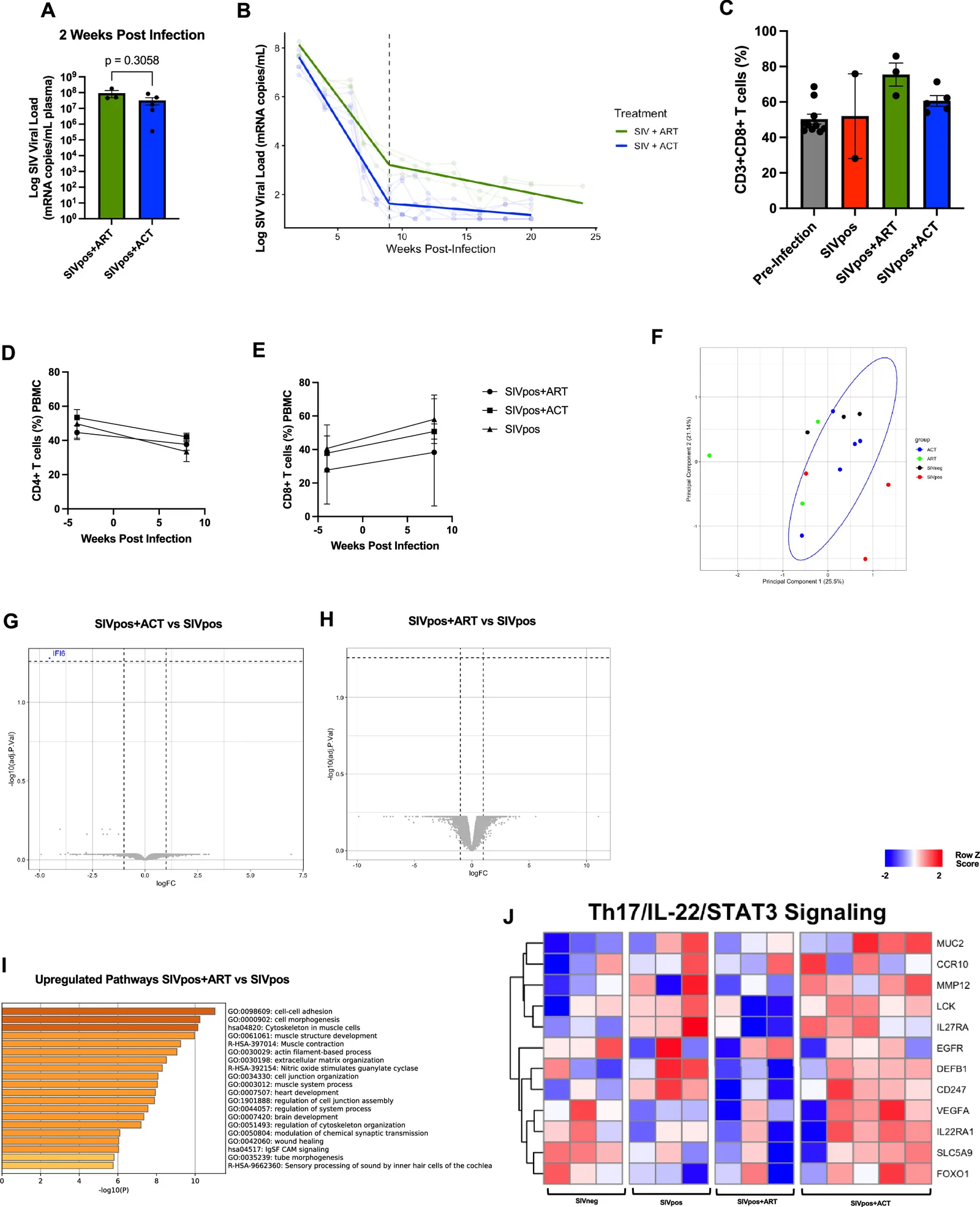
IL22/STAT3 signaling increased with combination of ART and 10-HSA treatment (ACT). (**A**) Peak viremia for ART (n = 3 biological replicates) and ART + 10-HSA/ACT (n = 5 biological replicates) treated animals. Data analyzed with unpaired one-way t-test. (**B**) Slope analysis of viral kinetics (n = 3 biological replicates SIVpos+ART, n = 5 biological replicates SIVpos+ACT). (**C**) CD8^+^ mucosal T-cell percentages from flow cytometry data at 2 weeks of treatment, SIVpos (n = 2 biological replicates), SIVpos+ART (n = 3 biological replicates), SIVpos+ACT (n = 5 biological replicates). Peripheral blood (**D**) CD4^+^ and (**E**) CD8^+^ percentages. SIVpos (n = 2 biological replicates), SIVpos+ART (n = 3 biological replicates), SIVpos+ACT (n = 5 biological replicates). (**F**) PCA plot from mucosal RNAseq data (n = 3 biological replicates for SIVneg, n = 3 biological replicates for SIVpos, n = 3 biological replicates for SIVpos+ART, n = 5 biological replicates for SIVpos+ACT. Volcano plots from (**G**) SIVpos+ART (n = 3 biological replicates) vs SIVpos (n = 3 biological replicates) and (**H**) SIVpos+ACT (n = 5 biological replicates) vs SIVpos (n = 3 biological replicates) groups. (**I**) Upregulated gene expression pathways in SIVpos+ART (n = 3 biological replicates) vs SIVpos (n = 3 biological replicates) animals. (**J**) IL22/STAT3 signaling gene expression z score graph. Data are presented as mean values +/- SEM. Source data

To determine whether the microbially derived 10-HSA enhances anti-retroviral efficacy in vivo, we performed another NHP study in which SIV-infected animals received a combination therapy consisting of both ART and 10-HSA, termed accelerated combination therapy (ACT), that was initiated at 6 weeks post SIV infection (Fig. 4a). Study groups included SIVpos + ACT (*n* = 5), SIVpos + ART (*n* = 3), untreated SIVpos (*n* = 2) and uninfected controls SIVneg (*n* = 3). The SIVpos + ACT animals exhibited substantially accelerated suppression of plasma viraemia, with marked reductions observed within a week of initiating ACT, in contrast to the more gradual decline of viral loads observed in SIVpos + ART animals (Fig. 4b,c). Untreated SIVpos animals maintained high viral loads (Fig. 4b). Importantly, peak viraemia before treatment initiation did not differ between SIVpos + ACT and SIVpos + ART groups, indicating that the accelerated viral suppression in the periphery was attributed to the inclusion of 10-HSA treatment (Extended Data Fig. 5a). Consistently, analysis of biphasic viral decay slope measurements showed a significantly steeper slope (*P* = 0.0264) in the first phase of viral suppression in ACT-treated animals compared with ART-alone controls (Extended Data Fig. 5b). We next assessed effects of ACT on immune reconstitution and mucosal integrity. ACT treatment induced an early rebound of colonic gut mucosal CD4^+^ T cells, accompanied by reduced mucosal CD8^+^ T cells, indicative of improved gut mucosal immune homeostasis (Fig. 4d and Extended Data Fig. 5c). In contrast, ART alone did not significantly restore CD4^+^ T-cell populations. At study endpoint, ACT-treated animals exhibited increased jejunal mucosal CD4^+^ T-cell frequencies relative to both ART-treated and untreated SIVpos animals, along with significant reduction in activated (HLA-DR^+^) CD4^+^ T cells, suggesting reduced immune activation (Fig. 4g,h). ACT, but not ART, restored gut epithelial barriers, as evidenced by continuous and organized ZO-1 distribution along the gut epithelium (Fig. 4e,f). In contrast, disruption of gut epithelial barriers was evidenced by significant reduction of ZO-1 protein continuity and visual density in both SIVpos and SIVpos + ART animals compared to SIVneg controls (Fig. 4e,f). A decrease in CD4^+^ T-cell percentages was seen among all groups with SIV infection (Extended Data Fig. 5d,e). The most pronounced effects of the viral infection as well as of ACT were evident in the gut mucosal microenvironment compared to the periphery.

To identify transcriptional pathways mediating the mucosal repair effects of ACT, we performed RNA-seq on gut tissues with the addition of another SIVpos control. PCA demonstrated that SIVpos + ACT animals clustered distinctly from both SIVpos and SIVpos + ART animals (Extended Data Fig. 5f). Differential gene expression analysis showed significant downregulation of the interferon-stimulated gene *IFI6* in SIVpos + ACT animals compared to SIVpos controls (Extended Data Fig. 5g). Comparison of SIVpos + ART with SIVpos animals showed no FDR-corrected significant alterations (Extended Data Fig. 5h). To gain further insight into ACT-mediated changes, we utilized a *P* value cut-off of ≤0.05 for pathway analysis. SIVpos + ACT animals showed increased expression of genes associated with mRNA metabolism, protein modification, immune regulation, cell activation and nuclear receptor signalling compared to SIVpos animals (Fig. 4i). PPARα signalling showed upregulation under the nuclear receptor signalling pathway (Fig. 4i). These same pathways were not enriched in only ART-treated animals (Extended Data Fig. 5i). Direct comparison between SIVpos + ACT and SIVpos + ART treatment groups further revealed upregulation of lipid metabolism pathways (Fig. 4j). ACT-treated animals showed increased expression of *PPARA* and lipid metabolism associated genes while SIVpos and ART-treated animals showed dysregulation of lipid metabolic programmes (Fig. 4k). Due to the marked reduction in viral loads and interferon signalling in ACT-treated animals, we sought to determine whether antiviral signalling and stress response were altered. SIVpos animals showed robust interferon and antiviral response gene signatures, while ART treatment only partially attenuated this pro-inflammatory antiviral signalling (Fig. 4l). In contrast, ACT treatment resulted in a pronounced suppression of antiviral and inflammatory signalling (Fig. 4l). SIV/HIV infection induces DNA damage through Vpr protein activity, hence SIVpos animals displayed elevated expression of DNA repair genes (Fig. 4m)^27^. While ART alone partially reduced this response, ACT-treated animals showed the most substantial normalization of DNA repair-related gene expression (Fig. 4m).

Building on our previous observation that 10-HSA enhances IL-22 signalling, we found that ACT-treated animals exhibited increased Th17/IL-22/STAT3 pathway activity compared to ART treatment, along with upregulation of mucosal defence genes including *CCR10* and *MUC2* (Extended Data Fig. 5j). To investigate mechanisms underlying rapid viral suppression with ACT, we utilized the J-Lat HIV latency cell model, which harbours a single HIV LTR-driven GFP reporter that is activated through NF-κB signalling by phorbol 12-myristate 13-acetate (PMA). Activation of the J-Lat HIV LTR induced GFP expression in 65% of the cells, whereas co-treatment with 10-HSA reduced it to 7% of the cells, indicating potent suppression of HIV LTR activation (Fig. 4n,o). PPARα activation suppresses NF-κB activity^28^. These findings suggest that 10-HSA may contribute to viral suppression by limiting NF-κB-dependent transcriptional activation of the viral promoter through PPARα activation.

### Protection of gut microbiota diversity following 10-HSA and ACT treatments

**Fig. 5:**
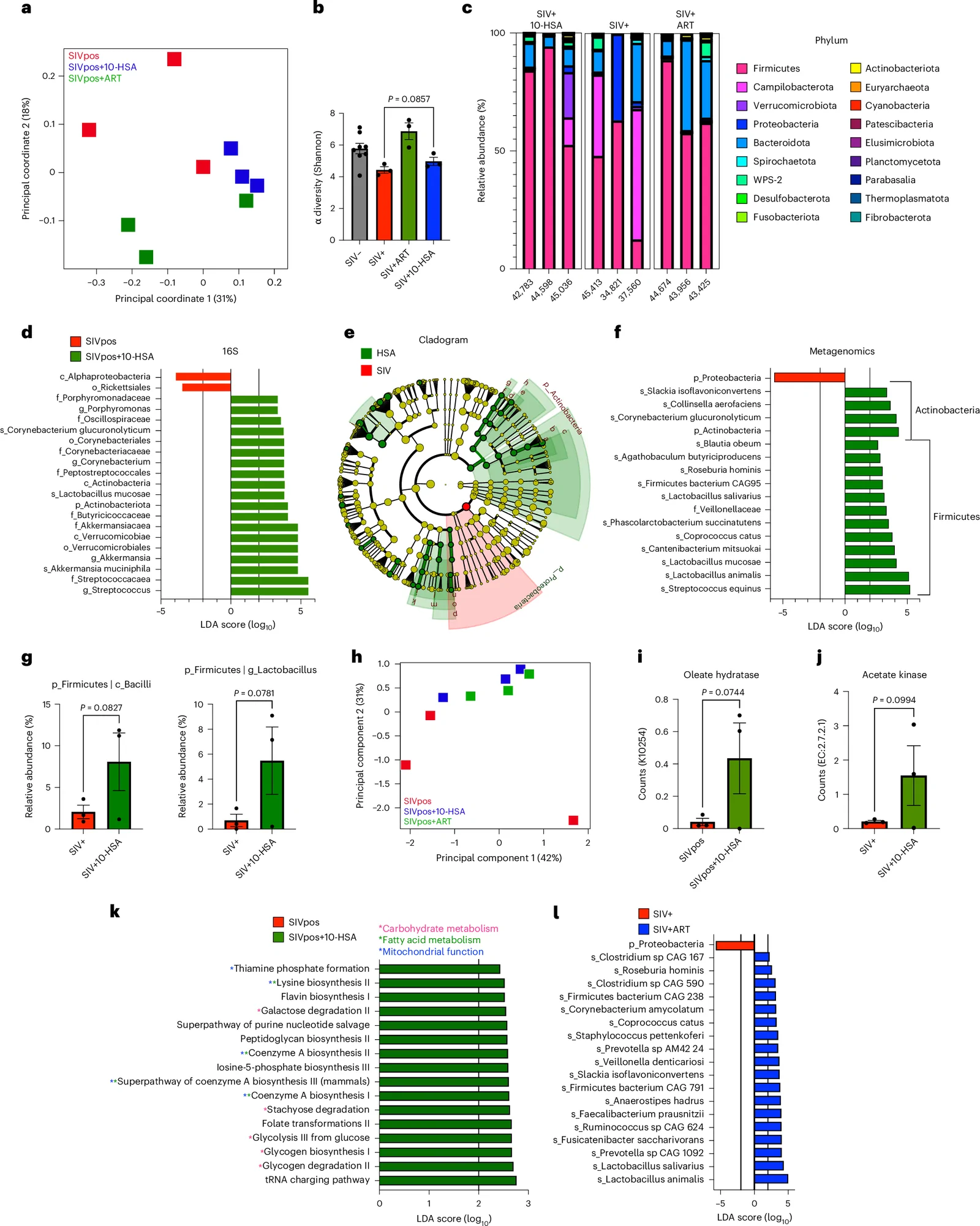
Restoration of gut microbiome following 10-HSA treatment in SIV infection. **a**, Microbiome changes detected by Bray–Curtis principal coordinates analysis (PCoA) plot of beta diversity between groups at the study endpoint (*n* = 3 biological replicates for SIVpos, SIVpos + ART, SIVpos + 10-HSA) based on 16S sequencing data. **b**, Microbiome alpha diversity as determined by Shannon index at the study endpoint based on the 16S sequencing data (*n* = 8 (SIVneg derived from pre-infection data), *n* = 3 (SIVpos), *n* = 3 (SIVpos + 10-HSA), *n* = 3 (SIVpos + ART) biological replicates). Data analysed using one-way unpaired *t*-test. **c**, Relative abundance of phyla across treatments based on 16S sequencing data. **d**, LEfSe-generated LDA plot comparing microbiomes from SIVpos vs SIVpos-HSA (*n* = 3 biological replicates per group) animals using 16S sequencing data. **e**, LEfSe cladogram generated from metagenomic data comparing gut microbiomes from SIVpos and SIVpos-HSA animals (*n* = 3 biological replicates per group). **f**, LDA plot from LEfSe analysis of metagenomic data focusing on taxa of Actinobacteria and Firmicutes based on SIVpos vs SIVpos-HSA comparisons (*n* = 3 biological replicates per group). **g**, Bacilli and *Lactobacillus* relative abundance in SIVpos and SIVpos-HSA groups as determined by metagenomic sequencing (*n* = 3 biological replicates per group). Data analysed using unpaired one-way *t*-test. **h**, PCA plot generated with limma.voom showing metagenomic-sequenced enzyme gene counts in the gut microbiome of SIVpos (*n* = 3), SIVpos-HSA (*n* = 3) and SIVpos + ART (*n* = 3) biological replicates. **i**, Counts per million of *OhyA* gene from metagenomic data between SIVpos and SIVpos-HSA animals (*n* = 3 biological replicates per group). Data analysed using one-way unpaired *t*-test. **j**, Counts per million of acetate kinase gene from metagenomic data between SIVpos and SIVpos-HSA animals (*n* = 3 biological replicates per group). Data analysed using one-way unpaired *t*-test. **k**, LEfSe LDA analysis identifying unique pathways between SIVpos and SIVpos-HSA (*n* = 3 biological replicates per group) gut microbiomes by metagenomic sequencing. **l**, LEfSe LDA analysis identifying unique taxa between SIVpos and SIVpos + ART (*n* = 3 biological replicates per group) groups by metagenomic sequencing. Data are presented as mean ± s.e.m.

**Extended Data Fig. 6:**
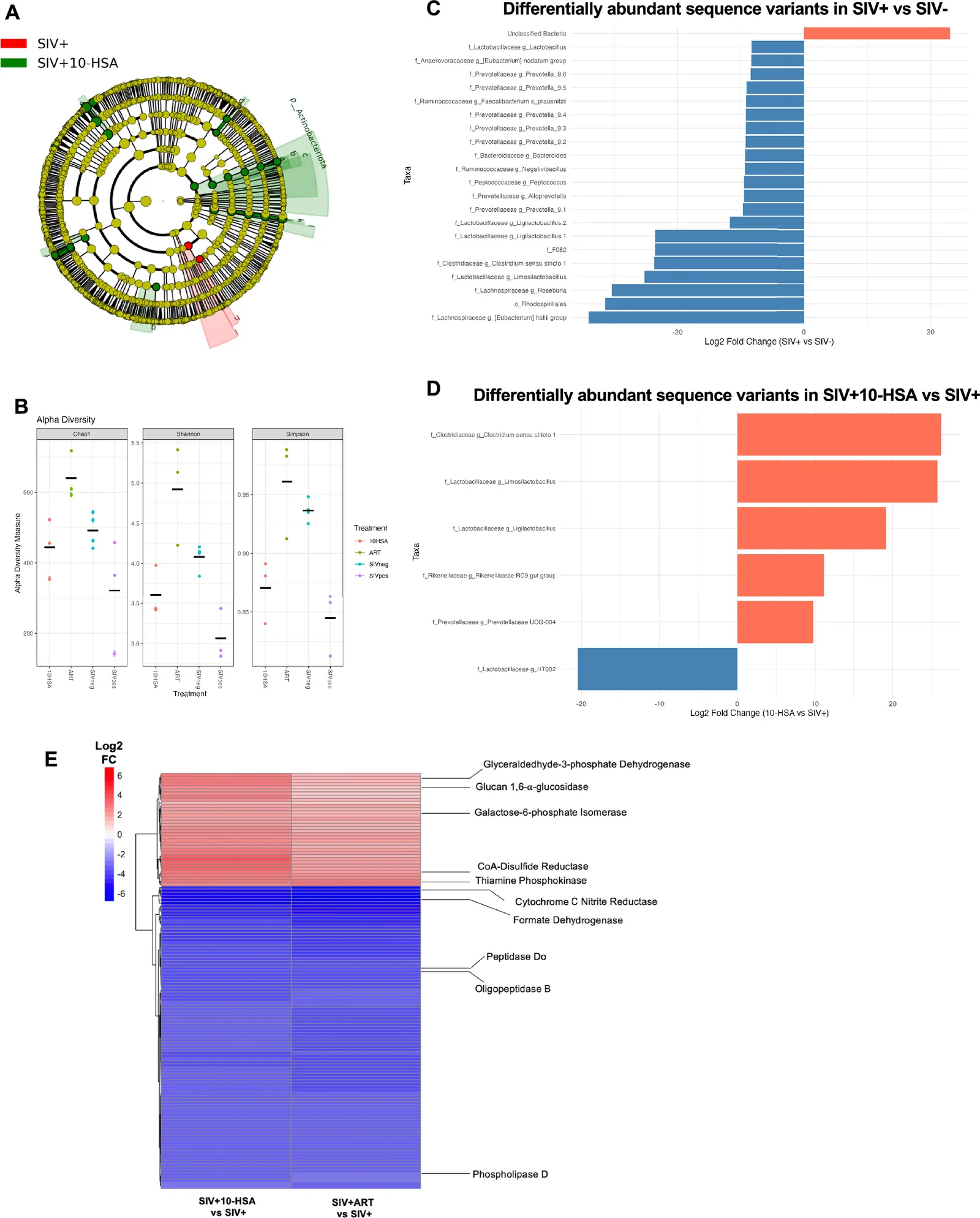
Increased prevalence of Firmicutes in 10-HSA treated animals detected by DEseq2 analysis of 16S data. (**A**) LEfSe cladogram output comparing SIVpos (n = 4 biological replicates) and SIVpos+10-HSA (n = 3 biological replicates) gut microbiome compositions from metagenomics data. (**Β**) Alpha diversity metrics (n = 3 biological replicates SIVpos, n = 4 biological replicates SIVneg, n = 3 biological replicates SIVpos+ART, n = 3 biological replicates SIVpos + 10-HSA). (**C**) SIVpos (n = 4 biological replicates) vs SIVneg (n = 3 biological replicates) significantly altered taxa from DEseq2 analysis. (**D**) SIVpos+10-HSA vs SIVpos (n = 3 biological replicates per group) significantly altered taxa from DEseq2 analysis. (**E**) Differential bacterial gene expression analysis from metagenomics data (n = 3 biological replicates SIVpos, n = 3 biological replicates SIVpos+ART, n = 3 biological replicates SIVpos+10-HSA).

We have shown previously that SIV infection rapidly promotes expansion of pathogenic bacteria in the gut microbiome through suppression of host pathogen recognition^29^. To investigate whether the microbially derived 10-HSA could modulate the gut microbiome, we performed 16S rRNA sequencing of colo-rectal swabs. Beta diversity analysis using Bray–Curtis dissimilarity indicated treatment-dependent differences in microbiome composition (Fig. 5a). Alpha diversity was increased in SIVpos-HSA animals compared to SIVpos controls, suggesting partial restoration of microbial communities (Fig. 5b). Taxonomic profiling showed that SIVpos-HSA-treated animals displayed a higher relative abundance of Firmicutes (that is, Bacillota) compared with either SIVpos or SIVpos + ART animals (Fig. 5c). Linear discriminant analysis (LDA) effect size (LEfSe) identified bacterial taxa enriched within each group (LDA score > 2) with clear separation between SIVpos and SIVpos-HSA animals (Extended Data Fig. 6a). SIVpos-HSA animals were enriched in the Firmicute taxa, namely *Streptococcus* and *Lactobacillus mucosae* as well as Verrucomicrobiales, Actinobacteriota (that is, Actinomycetota) and Butyricicoccacaea taxa (Fig. 5d). In contrast, SIVpos animals showed selective enrichment of Alphaproteobacteria, an opportunistic pathogenic taxon associated with HIV and SIV infections (Fig. 5d)^30,31^. To address potential methodological bias, we validated these findings using an independent DEseq2 pipeline^32^. Alpha diversity metrics from DADA2 confirmed the increased diversity due to 10-HSA treatment (Extended Data Fig. 6b). SIVpos animals showed decreased abundance of Lachnospiraceae and Lactobacillaceae families compared to SIVneg animals, whereas 10-HSA treatment significantly (*P*_adj_ < 0.05) increased *Limosilactobacillus* and *Ligilactobacillus* genera compared to SIVpos animals, confirming expansion of beneficial Firmicutes due to ACT treatment (Extended Data Fig. 6c,d).

Metagenomic sequencing was performed to further define microbiome composition and function. LEfSe analysis confirmed the enrichment of phylum Proteobacteria (Pseudomonadota) in SIVpos animals, while SIVpos-HSA animals had increased Actinobacteria and Firmicute populations (Fig. 5e,f). At the species level, *Lactobacillus* species and *Blautia obeum* were significantly enriched in SIVpos-HSA animals compared to SIVpos animals, with a total of 12 Firmicutes and 3 Actinobacteria species (Fig. 5f). The class of bacilli and genus *Lactobacillus* showed increased relative abundance following 10-HSA treatment (Fig. 5g). Metagenomic functional profiling identified 135 bacterial enzyme genes that were differentially abundant (FDR < 0.15, *P* < 0.05) in SIVpos-HSA and SIVpos animals. The PCA plot of metagenomic analysis showed clustering of SIVpos-HSA and SIVpos + ART animals, indicating partially overlapping functional profiles (Fig. 5h and Extended Data Fig. 6e). SIVpos-HSA animals showed increased expression of genes encoding thiamine phosphokinase, CoA-disulfide reductase and carbohydrate metabolism enzymes compared to SIVpos animals (Extended Data Fig. 6e). Genes encoding pro-inflammatory bacterial enzymes including cytochrome C nitrate reductase, formate dehydrogenase, peptidase DO, oligopeptidase B and phospholipase D were significantly downregulated in SIVpos-HSA animals compared to SIVpos animals (Extended Data Fig. 6e). Further analysis of metagenomic data revealed substantially increased abundance of the oleate hydratase gene *OhyA* in 10-HSA-treated animals compared to SIVpos animals (Fig. 5i). OhyA catalyses the conversion of oleic acid to 10-HSA^33^. Acetate kinase expression was also increased with 10-HSA treatment (Fig. 5j).

SIVpos-HSA animals showed increased thiamine phosphate and coenzyme A biosynthesis pathways compared to SIVpos controls (Fig. 5k). Although metagenomic gene expression profiles of SIVpos-HSA and SIVpos + ART groups were generally similar, SIVpos + ART animals showed increased abundance of *Lactobacillus* species *L. salivarus* and *L. animalis* but notably not *L. mucosae* while remaining enriched with *Prevotella* and *Clostridium*, suggesting only partial restoration of gut microbiome (Fig. 5l). We also investigated changes in the diversity of oral microbiomes in SIVpos-HSA and SIVpos animals. The oral mucosal microbiome of SIVpos-HSA animals displayed enrichment for Firmicutes (Supplementary Fig. 2).

**Fig. 6:**
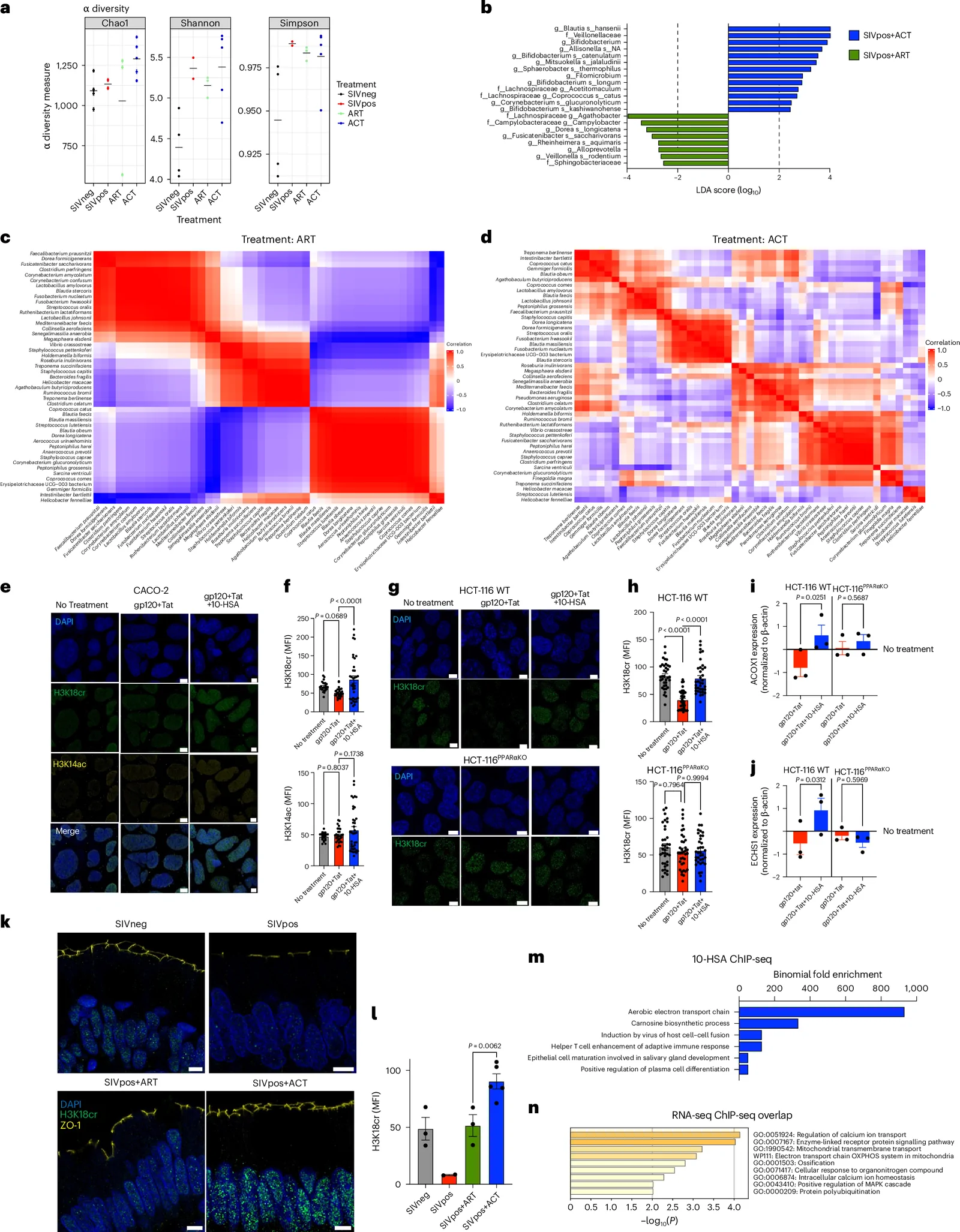
PPARα activation promotes mitochondrial energy metabolism gene expression through host epigenetic remodelling. **a**, Chao1, Shannon and Simpson alpha diversity metrics for SIVneg (*n* = 4 biological replicates), SIVpos (*n* = 2 biological replicates), SIVpos + ART (*n* = 3 biological replicates) and SIVpos + ACT (*n* = 5 biological replicates). **b**, LEfSe analysis of SIVpos + ACT (*n* = 5 biological replicates) treated animals vs SIVpos + ART (*n* = 3 biological replicates) treated animals. **c**,**d**, Pearson’s correlation of species-level data from ART (**c**) (*n* = 3 biological replicates) and ACT (**d**) (*n* = 5 biological replicates) treated animals. **e**,**f**, Histone crotonylation and acetylation immunofluorescence representative images in Caco-2 cells (**e**) with semi-quantification (**f**) (*n* = 24 cells per treatment group) with analysis using one-way ANOVA. Scale bar, 5 μm. **g**,**h**, Histone crotonylation and acetylation immunofluorescence representative images in HCT-116 WT and PPARα ΚΟ cell lines (**g**) with semi-quantification with analysis using one-way ANOVA (**h**) (*n* = 30 cells per treatment). Scale bar, 5 μm. **i**,**j**, Expression of ACOX1 (**i**) and ECHS1 (**j**) in HCT-116 WT and PPARα KO cell lines with viral proteins and 10-HSA (*n* = 3 biological replicates per group). Data analysed using unpaired one-way *t*-test. **k**,**l**, Representative micrographs of the jejunal gut epithelium (**k**) (*n* = 3 (SIVneg), *n* = 2 (SIVpos), *n* = 3 (SIVpos + ART), *n* = 5 (SIVpos + ACT) biological replicates) stained for ZO-1 and H3K18cr with semi-quantification (**l**) (*n* = 3 (SIVneg), *n* = 2 (SIVpos), *n* = 3 (SIVpos + ART), *n* = 5 SIVpos + ACT) biological replicates), analysed using one-way unpaired *t*-test. Scale bar, 5 μm (**k**). **m**, Pathway analysis of enriched genes from ChIP-seq data set in cells treated with 10-HSA. **n**, Overlap of SCDM RNA-seq from cells treated with viral proteins and 10-HSA with ChIP-seq data set in cells treated with 10-HSA. Data are presented as mean ± s.e.m. Source data

**Extended Data Fig. 7:**
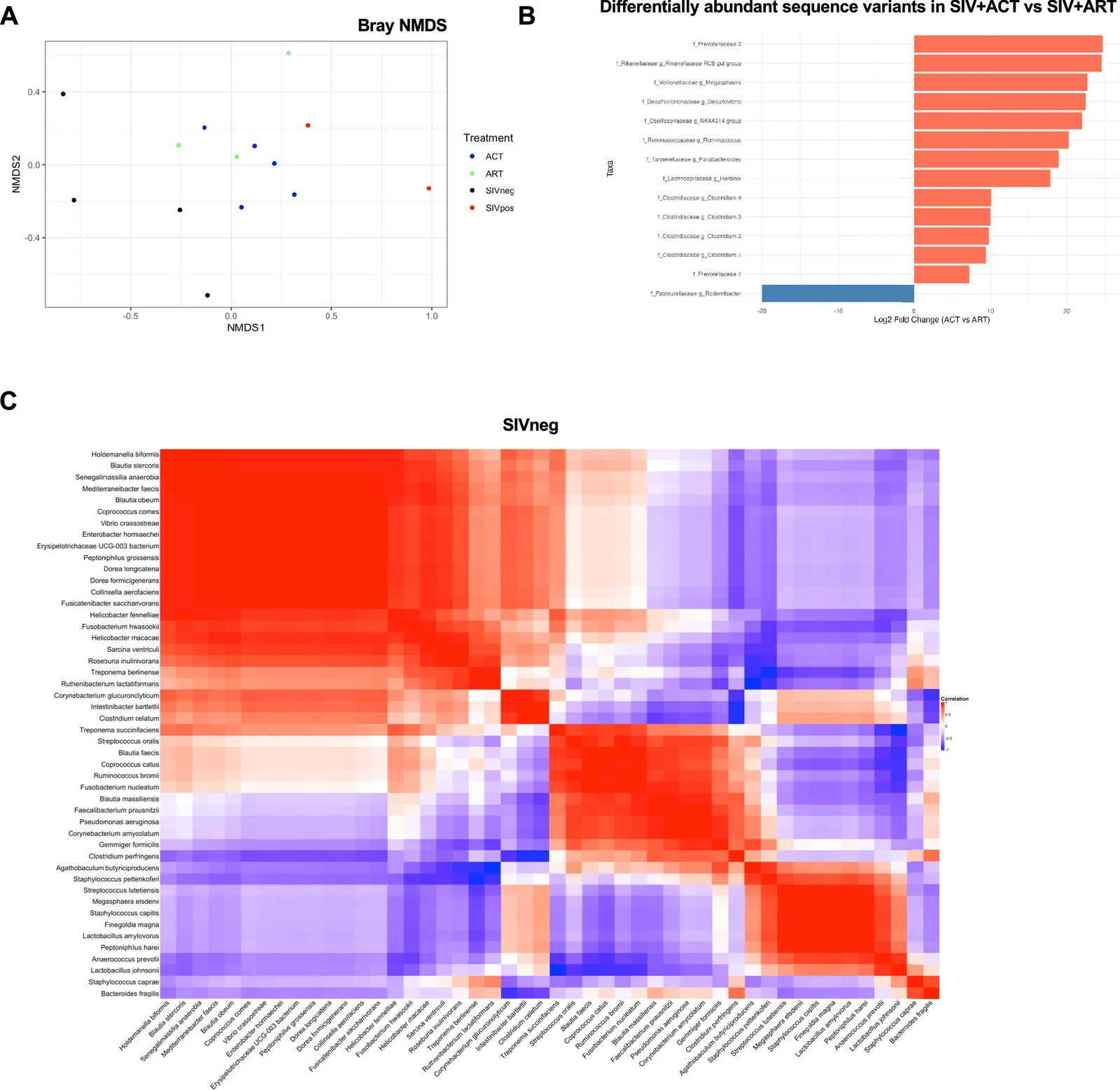
Increased diversity of gut microbial communities following ACT. (**A**) Bray-Curtis Beta diversity (n = 3 biological replicates SIVneg, n = 3 biological replicates SIVpos, n = 3 biological replicates SIVpos+ART, n = 5 biological replicates SIVpos+ACT). (**B**) DESeq2 analysis of 16S data (n = 5 biological replicates SIVpos+ACT, n = 3 biological replicates SIVpos+ART). (**C**) Pearson’s correlation of species level 16S data in SIVneg (n = 3 biological replicates) animals.

Finally, we compared microbiomes of ACT and ART-treated animals. ACT-treated animals exhibited greater alpha diversity and altered beta diversity compared with SIVpos and SIVpos + ART animals (Fig. 6a and Extended Data Fig. 7a). ACT treatment was associated with increased microbiome diversity and substantial enrichment of Lachnospiraceae, *Blautia* and *Bifidobacterium* compared to ART alone (Fig. 6b). One of the most significantly (LDA > 2) increased genera was *Blautia* which validated our previous findings (Fig. 6b). ACT-treated animals showed increased *Coprococcus catus* abundance which confirmed our findings from animals receiving 10-HSA treatment only (Figs. 5f and 6b). Both DEseq2 and LEfSe methods of analysis showed consistent effects of 10-HSA on the expansion of Lachnospiraceae (Extended Data Fig. 7b). Pearson’s correlation detected unique clustering of *Lactobacillus amylovorus, L. johnsonii*, *Blautia obeum*and *B. faecis* species in ACT-treated animals—a pattern not observed in SIVpos + ART or SIVneg animals (Fig. 6c,d and Extended Data Fig. 7c). Collectively, these findings demonstrate that 10-HSA alone and in combination therapy ACT restore gut microbiome diversity and promote expansion of beneficial taxa.

### Histone crotonylation through a PPARα-mediated mechanism

**Extended Data Fig. 8:**
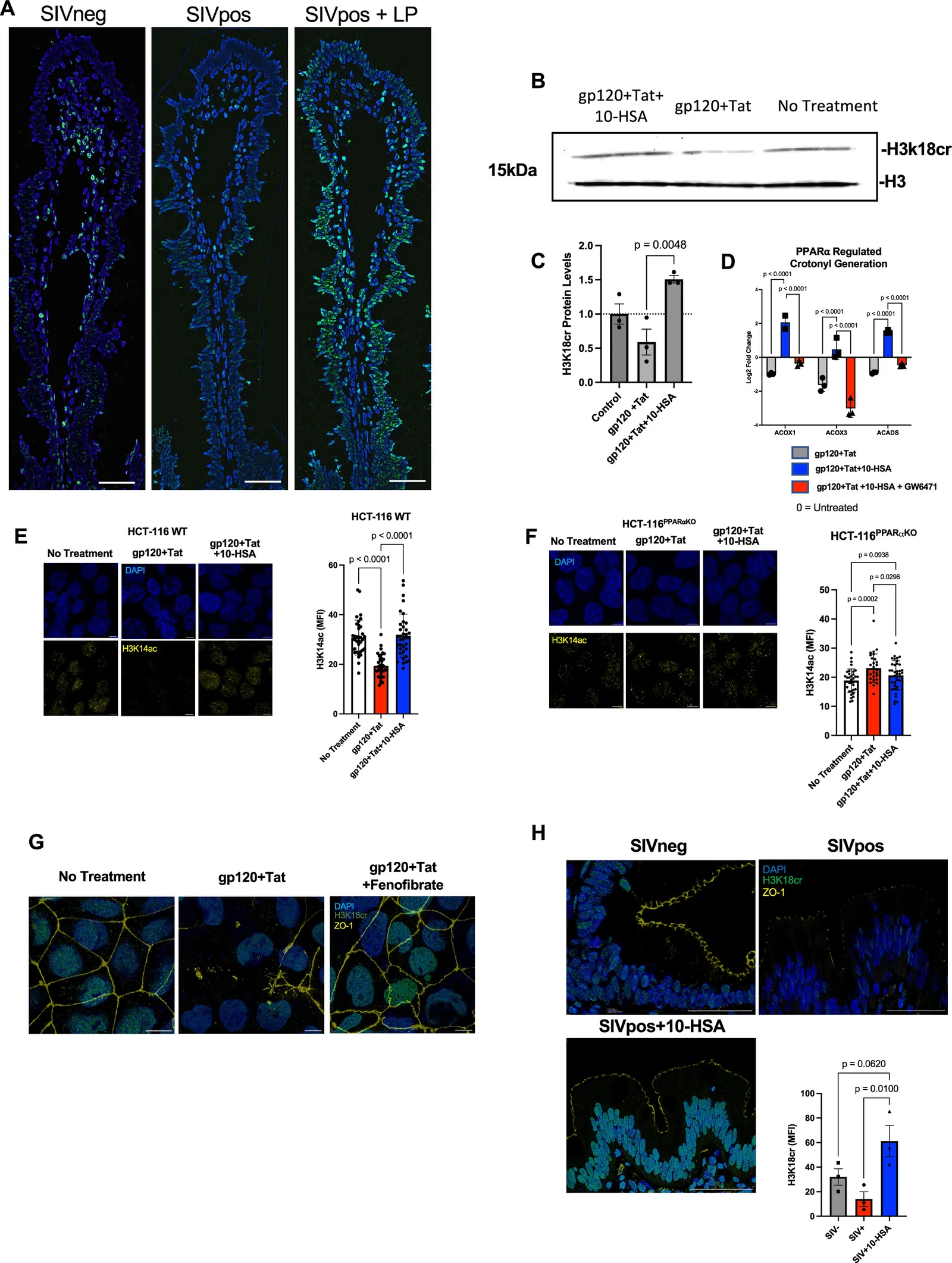
PPARα activation induces histone crotonylation and epithelial repair in vivo. (**A**) Representative images of SIVneg (n = 3 biological replicates), SIVpos (n = 10 biological replicates), and SIVpos+LP (n = 7 biological replicates) treated animals showing H3K18cr. Scale bar = 50μm. (**B**) Western blot with (**C**) quantification showing H3K18cr levels in Caco2 cells (n = 3 biological replicates per treatment) after viral protein exposure and 10-HSA treatment. Data analyzed by unpaired one-way t-test. (**D**) Expression of PPARα regulated crotonyl group producing enzymes associated with fat metabolism (n = 3 biological replicates per group). Data analyzed by mixed-effects analysis. Acetylation (H3K14ac) representative images and semi-quantification in (**E**) HCT-116 WT and (**F**) HCT-116 PPARα KO cells (n = 35 cells per treatment) after treatments. Experiment run in duplicates. Scale bar = 5μm. Data was analyzed by one-way ANOVA. (**G**) Fenofibrate treated Caco2 (n = 3 biological replicates) cells stained for ZO-1 and H3K18cr. (**H**) Representative images of SIVneg, SIVpos, and SIVpos+10-HSA (n = 3 biological replicates per group) treated animals showing ZO-1 and H3K18cr. Scale bar = 25 micron. Data was analyzed by one-way ANOVA. Data are presented as mean values +/- SEM. Source data

We hypothesized that the rapid repair of the epithelial barriers following *L. plantarum* treatment into SIV-infected gut was mediated in part through histone lysine crotonylation (Kcr)^34^. Immunohistochemical analysis showed increased Kcr in the SIV-inflamed gut tissue exposed to *L. plantarum* as compared to the untreated SIV-inflamed gut (Extended Data Fig. 8a). We examined the effects of HIV antigen exposure and the microbially derived 10-HSA treatment on histone crotonylation in Caco-2 cells in vitro. Viral antigen exposure decreased H3K18cr levels compared to unexposed controls with minimal impact on H3K14ac levels, while 10-HSA significantly increased H3K18cr levels (Fig. 6e,f). Western blot analysis confirmed increased H3K18cr levels in cells following 10-HSA treatment (Extended Data Fig. 8b,c). We next sought to determine whether histone crotonylation was in part regulated by PPARα signalling. Crotonyl-CoA production is partially controlled by PPARα-regulated enzymes *ACOX1, ACOX3, ACADS* and *ECHS1* (ref. ^35^). The expression of PPARα-regulated genes was significantly increased by 10-HSA, while pharmacological inhibition of PPARα by GW6471 suppressed the expression of these genes even in the presence of 10-HSA (Extended Data Fig. 8d). In wild-type HCT-116 cells, viral antigen exposure reduced H3K18cr and H3K14ac levels, both of which were rapidly restored within 30 min of 10-HSA treatment (Fig. 6g,h and Extended Data Fig. 8e). In contrast, HCT-116^PPARαKO^ cells exhibited no change in H3K18cr levels in response to viral antigens or 10-HSA and no alterations in the expression of *ACOX1* and *ECHS1* genes (Fig. 6g,h(bottom),i,j). Histone acetylation was decreased in 10-HSA-treated HCT-116^PPARαKO^ cells (Extended Data Fig. 8f). WT HCT-116 cells showed suppression of these genes following HIV antigen exposure and recovery with 10-HSA treatment (Fig. 6i,j). To further validate the role of PPARα in this process, known PPARα ligand, fenofibrate, was used in the Caco-2 cell model. It induced repair of the ZO-1 tight junction structure and epithelial integrity in HIV antigen-exposed epithelial cells which was associated with increased Kcr (Extended Data Fig. 8g)^9,36^. We next investigated the role of Kcr in gut barrier repair in vivo. SIVpos animals showed reduction in H3K18cr levels in the gut epithelium compared to healthy controls (Fig. 6k,l and Extended Data Fig. 8h). ACT and 10-HSA alone increased epithelial H3K18cr levels (Fig. 6k,l and Extended Data Fig. 8h). SIVpos + ACT animals had heightened H3K18cr levels compared to SIVpos + ART animals (Fig. 6k,l). Expression of genes regulating crotonyl-CoA synthesis was increased in 10-HSA-treated animals (Supplementary Fig. 3a). We found no major alterations in histone acetylation patterns (Supplementary Fig. 3b).

**Extended Data Fig. 9:**
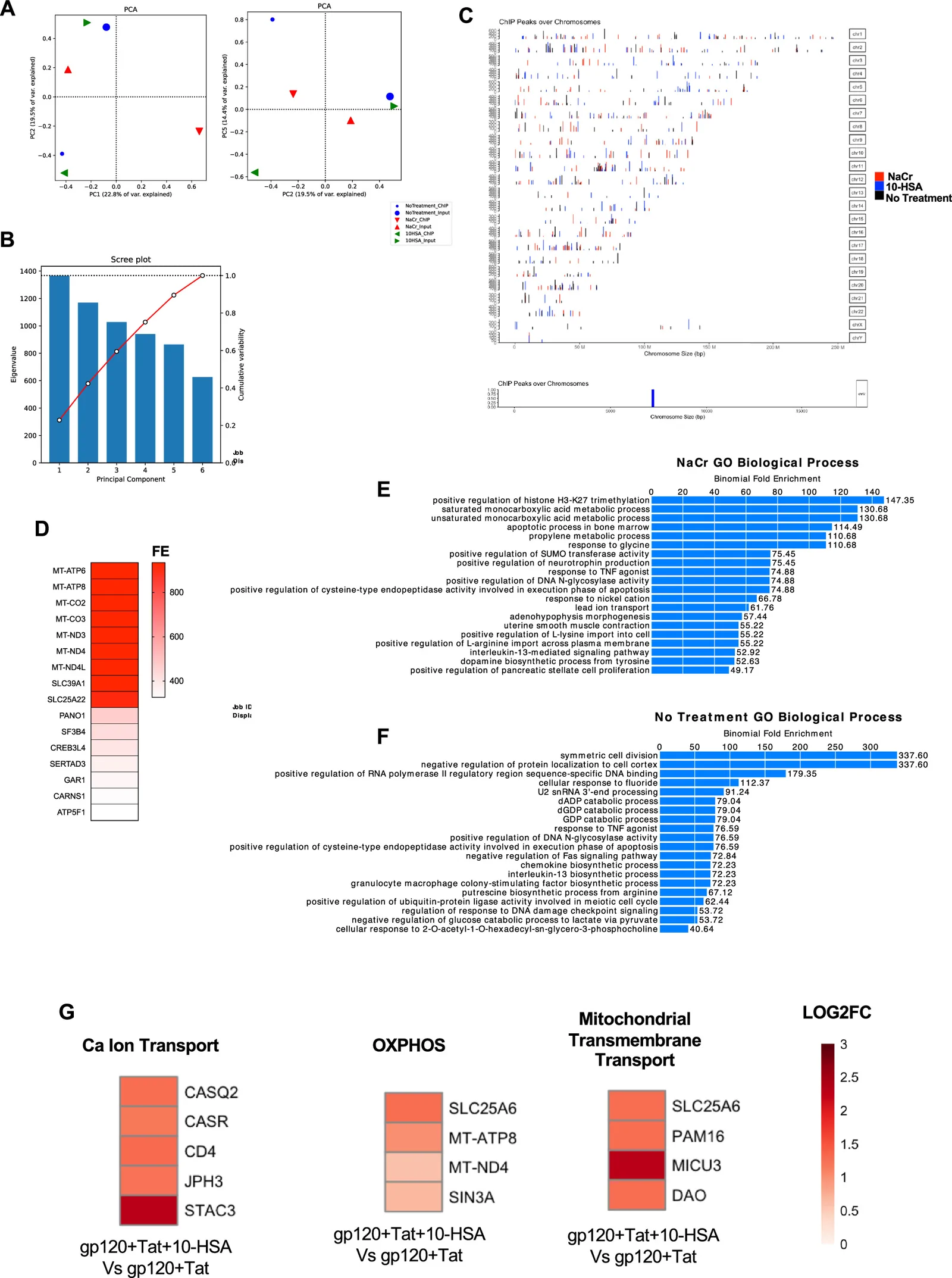
ChIP-seq analysis of histone crotonylation in 10-HSA or Sodium Crotonate treated Caco2 epithelial cells. (**A**) PCA plots. (**B**) SCREE plot. (**C**) Visualization of ChIP peaks spread across the human genome. (**D**) Top 16 enriched genes due to 10-HSA treatment (n = 20 million cells per treatment). (**E**) Pathway analysis of enriched genes in NaCr (n = 20 million cells per treatment) treated cells. (**F**) Pathway analysis of enriched genes in No treatment (n = 20 million cells per treatment) cells. (**G**) RNAseq analysis of significantly increased gene expression due to 10-HSA which overlaps with ChIP-seq data.

To determine the genomic landscape of crotonylation, we performed chromatin immunoprecipitation sequencing (ChIP-seq) with a pan-crotonyl lysine antibody in Caco-2 cells treated with 10-HSA and compared the patterns with cells treated with sodium crotonate (NaCr) and untreated controls. PCA analysis and peak mapping revealed distinct and treatment-specific Kcr profiles across treatment groups (Extended Data Fig. 9a–c). Visualization of PC5 and PC2 showed separation of all three treatment groups along PC5 (Extended Data Fig. 9a). Peak calling by Genrich showed differences in enriched genomic crotonyl signatures across treatment groups (Extended Data Fig. 9c). In 10-HSA-treated cells, a unique enrichment of crotonyl peaks was seen on chromosomes 4, 22, X and mitochondrial DNA, and was associated with genes involved in electron transport chain function and ATP synthesis (Extended Data Fig. 6m, Fig. 9C–F)^37^. In contrast, NaCr treatment did not lead to enrichment of mitochondrial or energy metabolism pathways (Extended Data Fig. 9e,f). Since NaCr increases cellular levels of Crotonyl-CoA from a non-metabolic source, it is reasonable to have non-specific broad effects of Kcr. Cross-referencing of RNA-seq data from HIV antigen-exposed SCDM following 10-HSA treatment with ChIP-seq data from 10-HSA-treated cells, we identified 54 significantly upregulated (FDR < 0.15, *P* < 0.05) genes associated with crotonyl binding sites (Fig. 6n). These genes were enriched in pathways regulating calcium ion transport, oxidative phosphorylation and mitochondrial transmembrane transport processes (Fig. 6n). Consistently, mitochondrial genes *ATP8* and *ND4* were upregulated following 10-HSA treatment in the context of viral antigen exposure (Extended Data Fig. 9g). Collectively, these findings identify a PPARα-regulated mechanism by which 10-HSA drives histone crotonylation and links epigenetic remodelling to mitochondrial and metabolic gene programmes during epithelial repair.

### PPARα signalling and crotonylation in long-term HIV non-progressors

**Extended Data Fig. 10:**
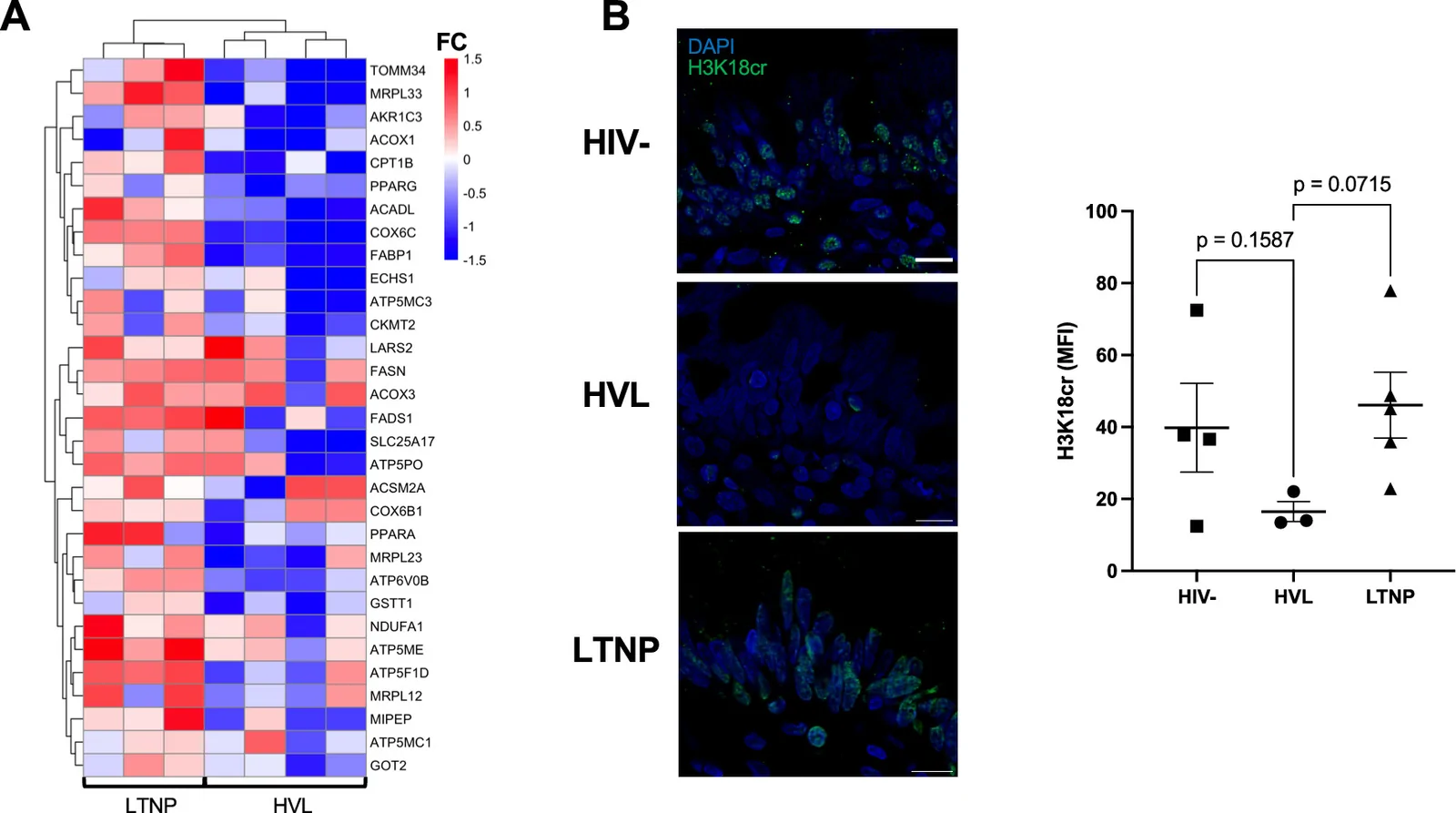
Increased histone crotonylation and PPARα signaling in gut tissues of Long-term HIV-positive Non-progressors. (**A**) PPARα and lipid metabolism gene expression of HVL (n = 4 biological replicates) and LTNPs (n = 3 biological replicates) from new analysis of previously published microarray. (**B**) Histone crotonylation from duodenal biopsies from HIVneg (n = 4 biological replicates), high viral load (HVL, n = 3 biological replicates), and long term non-progressors (LTNP, n = 4 biological replicates) with semi-quantification. Scale bar = 10 micron. Data was analyzed by one-way ANOVA. Data are presented as mean values +/- SEM. Source data

The gut mucosal landscape in long-term HIV non-progressors (LTNP) shows preservation of gut epithelial barrier integrity, mucosal CD4^+^ T-cell prevalence and gut microbiome diversity^38–40^. To investigate whether metabolic and epigenetic pathways contribute to this phenotype, we analysed gut biopsies of LTNP individuals and compared them to those from therapy-naïve individuals with chronic HIV infection (HVL) as well as HIVneg healthy controls. Immunohistochemical analysis revealed elevated Kcr levels within the gut epithelium of LTNPs compared to HVL individuals (Extended Data Fig. 10a,b). Gene expression analysis of human gut biopsies from our previously reported datasets revealed increased expression of PPARα-regulated *ACOX1, ACOX3* and *ACADS* in LTNP compared to HVL and HIV-negative healthy controls (Extended Data Fig. 10a)^40^. Increased expression of mucosal genes responsible for mitochondrial ATP synthesis and fatty acid metabolism was detected in LTNP compared to HVL, indicating PPARα activation (Extended Data Fig. 10a). Collectively, our findings suggest that the gut mucosal resilience in LTNP is associated with PPARα signalling and increased Kcr. Notably, these data mirror those observed in 10-HSA and ACT-treated SIVpos macaques, suggesting a conserved metabolic-epigenetic programme underlying gut epithelial barrier maintenance during viral infection.

## Discussion

Our study identified the microbially derived 10-HSA as a biotherapeutic metabolite produced during the initial response of *L. plantarum* to the SIV-inflamed gut. Combining 10-HSA with anti-retroviral therapy (ACT) integrates antiviral activity with gut mucosal repair, addressing and overcoming a key limitation of ART, which does not resolve gut mucosal disruption^9^. Given that gut inflammation and epithelial disruption are strong predictors of HIV-associated morbidity, strategies that restore mucosal integrity are of paramount importance^41,42^. Both 10-HSA and ACT treatments promoted recovery of the gut epithelial barrier structure and function, reduced mucosal inflammation and restored the microbiome, indicating enhanced mucosal homeostasis. 10-HSA treatment promoted gut microbial diversity and expansion of Firmicutes populations in both NHP studies, which is consistent with our previous findings in a distinct gut–liver injury model^43^. We reason that gut mucosal repair and renewal during ACT enabled the restoration of microbiome by providing a supportive gut microenvironment. Strikingly, ACT accelerated viral suppression while promoting concurrent mucosal repair, underscoring the central role of mucosal immunity in viral control. Recent anti-PD1 and anti-CTLA4 treatments for HIV cure studies have not been effective in viral eradication and showed toxic side effects^44^. These findings emphasize that revitalizing mucosal immunity may present a more effective strategy for improving viral remission than reactivating exhausted host immune cells. Our data provide new opportunities for enhancing the strategies for durable viral silencing and clearance.

Our study identified PPARα as a central node linking 10-HSA to epithelial repair. PPARα signalling is impaired in HIV and SIV infections, and we show that its activation is associated with gut mucosal resilience in long-term non-progressors^9,40^. LTNPs displayed heightened PPARα signalling and downstream transcriptional activity relative to therapy-naïve individuals, supporting a role for PPARα activation in long-term host antiviral defence. Using genetic and pharmacological approaches, we demonstrate that HIV antigens suppress PPARα signalling, disrupt metabolic programmes and impair epithelial repair, whereas 10-HSA restores PPARα activity and downstream transcriptional networks. PPARα activation also drives histone crotonylation, linking fatty acid metabolism to epigenetic regulation. 10-HSA-induced histone crotonylation was associated with transcriptional activation of mitochondrial and metabolic pathways, suggesting an epigenetic mechanism by which metabolic inputs remodel chromatin to support gut epithelial regeneration. The absence of this response in PPARα-deficient cells, together with increased crotonylation in LTNPs, underscores the functional importance of this pathway in host defence. Previous publications support the activation of PPARα by 10-HSA^45–47^.

In addition, we posit that the microbially derived 10-HSA significantly reduced SIV viral load in ACT-treated animals through PPARα activation and subsequent suppression of NF-κB. Our data support the use of 10-HSA as a combination therapeutic to rapidly suppress viral replication when combined with ART. There is an urgent need to identify novel strategies for durable HIV suppression and deep silencing to reduce the dependence on life-long ART through leveraging host immunity^48,49^. This study has some limitations. Namely, pharmacokinetic assessment of 10-HSA was not performed. In addition, the use of the non-human primate model results in low sample sizes; however, the consistency in results across two independent NHP studies demonstrates the reproducibility of our findings. Collectively, our findings define a microbiome–metabolite–PPARα–epigenetic axis that coordinates mitochondrial function, microbial ecology and epithelial repair in the context of viral inflammation. Our data position 10-HSA and PPARα activation as therapeutic strategies to restore gut barrier integrity, enhance mucosal immunity, rebalance the microbiome and mitigate HIV-associated disease progression while augmenting ART efficacy for durable viral remission and deep silencing.

## Methods

All research in this paper complies with all relevant ethics regulations set in place by the UC Davis Institutional Animal Care and Use Committee (IACUC) and Institutional Review Board (IRB).

### Rhesus macaque study design and sample collection

Twelve rhesus macaques were used for the first study reported. Three males aged 7–10 years provided SIVneg controls. Three males aged 7–10 years provided SIVpos controls. Three males aged 7–10 were infected with SIV and treated with 10-HSA. One female and two males were infected with SIV and treated with ART. For the second study reported, 12 more rhesus macaques were used. Three males aged 7–10 provided SIVneg controls. Two males aged 5–7 years provided SIVpos controls. Two females aged 4–7 years provided ART treatment controls. Five animals were administered ACT treatment (two females and three males aged 5–7 years). ART or ACT treatment was initiated at 6 weeks post infection until 20 weeks, at which point the study concluded. Rhesus macaques (*Macaca mulatta*) were obtained from and housed at the California National Primate Research Center (CNPRC). For the first study reported, three animals selected at random were pre-treated with 10 mg kg^−1^ per day of 10-HSA orally daily for 1-week pre-SIV infection. CNPRC staff ensured that animals consumed the full dose daily. All SIVpos animals were intravenously (i.v.) infected with 1,000 tissue culture infectious dose 50% (TCID_50_) of SIVmac251 on day zero. At 7 weeks, the dosage was increased to 25 mg kg^−1^ day^−1^ for the remainder of the first study. Initial dosage was low to allow for observation of potential negative side effects. None were seen so dosage was increased in accordance with the IACUC protocol. ART treatment consisted of 1 ml kg^−1^ body weight of 2.5 mg ml^−1^ dolutegravir (DTG) free base, 5.1 mg ml^−1^ tenofovir disoproxil fumarate (TDF) and 40 mg ml^−1^ emtricitabine (FTC) in 15% kleptose water administered subcutaneously daily. Blood draws were performed weekly for each animal and complete blood count (CBC) and fluorescence activated cell sorting (FACS) were conducted. Necropsy was conducted at week 12 where gut tissue, spleen, lymph tissue, lung tissue, and rectal and buccal swabs were obtained for further processing and analysis.

ART (*n* = 3) and ACT (*n* = 5) treated animals were i.v. infected with 1,000 TCID_50_ of SIVmac251 and received the same ART regimen as above. SIVpos with ART animals from the first study reported were necropsied after 18 weeks of ART. For the second study, reported animals were given ART at the same doses as above with 40 mg kg^−1^ day^−1^ of oral 10-HSA starting at 6 weeks post infection until endpoint. For the previously published non-human primate study investigating the therapeutic impact of *L. plantarum*, 16 rhesus macaques were enrolled. Of these, 12 macaques were i.v. infected with 1,000 TCID_50_ SIVmac251 for 10 weeks. Intestinal loop ligation was performed (4–6-cm-long loops) with 1 cm spacer loops. Loops were injected with 1 ml sterile deMan, Rogosa and Sharpe (MRS) culture broth or sterile MRS culture broth containing 1 × 10^9^ colony-forming units of *L. plantarum* NCIMB8826-MM24. MRS broth and *L. plantarum* remained within the intestinal loop for 5 h, after which tissue was collected for histopathological, transcriptomic and metabolomic assessment. SIVpos animals receiving ART and 10-HSA were administered 40 mg kg^−1^ day^−1^ of 10-HSA with the same ART protocol as above. Colo-rectal biopsies were taken at pre-infection timepoint and 8 weeks post infection to determine T-cell populations in colonic mucosa. This study included two more SIVpos animals which was raised to three for sequencing analysis. Viral load analysis of peripheral blood plasma was conducted at the Quantitative Molecular Diagnostics Core at Frederick National Laboratory using the standard sensitivity assay using qPCR to amplify the SIVgag RNA sequence. This study was performed in accordance with the recommendations and guidelines of the Public Health Services Policy on Humane Care and Use of Laboratory Animals. All procedures were performed in accordance with the protocols approved by the IACUC of the University of California Davis. The study reporting the effects of *L. plantarum* on the gut epithelial lining and production of 10-HSA was approved by UCD IACUC #21582. The two studies reporting the effect of 10-HSA in the SIV-infected non-human primate model in this paper were approved by UCD IACUC #22832. Pre-approved sedatives, anaesthetics and analgesics were used during animal handling, surgical procedures and at necropsy to minimize pain or discomfort to the animals. Animals were euthanized in accordance with the American Veterinary Medical Association Guidelines for the Euthanasia of Animals.

### Cells and treatments

Caco-2 cells (ATCC HTB-37), small-intestinal epithelial stem cell-derived epithelial monolayers from human patient samples, J-Lat 10.6 cells (Cellosaurus, CVCL_8281) and HCT-116 cells (Ubigene, YKO-H592) were used in the study. Caco-2 cells were cultured according to manufacturer recommendations in minimal essential media containing 20% HI-FBS and 1% penicillin/streptomycin. J-Lat 10.6 cells were cultured according to manufacturer recommendations in RPMI medium supplemented with 10% HI-FBS and 1% penicillin/streptomycin. HCT-116 cells were cultured according to manufacturer recommendations in McCoy’s 5A media supplemented with 10% HI-FBS and 1% pen/strep. Stem cell-derived monolayers (provided by the Phillip Smith Laboratory, University of Alabama at Birmingham) were cultured in DMEM media (Gibco) with 5% mouse fibroblast LWRN conditioned media (manufactured in-house), 1% nicotinamide (Sigma), 1% Fungizone (Fisher), 0.1% Y-27632 (Fisher) and 0.1% gentamicin (Sigma). Proper approvals and consent were given to the laboratory of Dr Phillip Smith at the University of Alabama for biopsies^50^. Cells were treated with gp120 (1 μg ml^−1^) and Tat (1.4 μg ml^−1^) to induce inflammatory conditions (NIH HIV reagent programme). 10-hydroxystearic acid (AstaTech) was water-bath sonicated in cell culture media to emulsify for 30 min and administered at 500 μM. Sodium crotonate (NaCr) was administered at 20 mM. Cells were treated with 480 nM PPARα antagonist GW6471 (Abcam) for 6 h. PPARα antagonist was resuspended in dimethyl sulfoxide (DMSO) according to manufacturer guidelines and diluted in cell culture media. J-Lat cells were cultured according to manufacturer guidelines. J-Lat cells were treated with 1 pg ml^−1^ PMA and 20 mM NaCr in RPMI supplemented with 10% FBS and 1% penicillin/streptomycin for 18 h to activate HIV-LTR GFP signal. Cells were co-treated with 500 μM 10-HSA. HCT-116^PPARαKO^ cells had PPARα knocked out by CRISPR-U at exon 5 of the PPARA-202 transcript according to manufacturer instructions (Ubigene). RT–qPCR for PPARα yielded no detectable signal after 30 cycles.

### Immunofluorescence

After described treatments for in vitro and ex vivo assays, media were removed, and cells were fixed for 15 min in ice-cold fresh 4% paraformaldehyde (PFA) and washed with 1× PBS then stored in 70% ethanol at 4 °C until immunofluorescence was performed. Cells were washed with 1× PBS and permeabilized with 0.1% Triton X-100 for 20 min at r.t. Cells were washed again with 1× PBS and blocked with 10% normal goat serum (NGS) in 1× PBS for 1 h at r.t. Blocking buffer was removed and primary antibody was diluted in blocking buffer at a 1:200 dilution ratio. Cells were placed overnight at 4 °C with primary antibody. The next day, cells were washed 5× with 1× PBS. Then secondary antibody was diluted in blocking buffer at 1:400 dilution ratio and placed on cells for 1 h at r.t. Cells were washed 5× with 1× PBS. Cells were stained with DAPI and mounted with Prolong Diamond Antifade mounting media (Invitrogen). Experiments were run in duplicate. Macaque gut tissue was fixed in ice-cold fresh 4% PFA immediately after removal from the animal for 24 h at r.t. Tissue was then placed in a plastic mounting cassette and submerged in 70% ethanol. Tissue was embedded in paraffin wax through the UC Davis Anatomic Pathology Service. Blocks were sectioned to 5 μm using a microtome and placed on charged slides to dry overnight. Slides were baked at 60 °C for 1 h the next day and then submerged in xylene and decreasing concentrations of ethanol in water to rehydrate. Slides were then permeabilized in 1% Triton X-100 for 20 min. Antigen retrieval was performed with pH 9 Tris-EDTA buffer (Agilent Dako) in a vegetable steamer. Slides were removed after 25 min and allowed to cool to r.t. Slides were then blocked with 10% NGS in 1× PBS for 1 h at r.t and incubated with primary antibody at a 1:200 dilution overnight at 4 °C. Slides were then washed (5×) with 1× PBS, and secondary antibody at a dilution ratio of 1:400 was placed on slides for 1 h at r.t. Nuclei were stained with DAPI and mounted with Prolong Diamond Antifade mounting media (Invitrogen). All quantification was performed blinded. Experiments were run in duplicate.

### Western blotting

Caco-2 cells were grown to 10^6^ density in 6-well culture plates. Cells were treated with viral antigens and 10-HSA as mentioned above. Cells were lysed with RIPA buffer containing 1× protease/phosphatase inhibitor cocktail (Thermo Fisher). Protein concentration was determined by bicinchoninic acid (BCA) assay. Proteins were heated at 95 °C for 30 min with 2.5% v/v 2-mercaptoethanol for reduction of disulfide bonds. Protein was loaded at 20 μg per lane in Novex 10% Tris-Glycine Plus WedgeWell gels (Invitrogen) and electrophoresed for 45 min at 200 V. Gel was removed from the plastic case and sandwiched in the Trans-Blot Turbo Transfer Pack utilizing the Trans-Blot Turbo Transfer System (0.2 μm PVDF, BioRad). MagicMarkXP Western Protein Standard (Thermo Fisher) was used for molecular weight identification. Blots were removed and trimmed with scissors, and blocked in 1× PBS-T with 5% v/v powdered skim milk for 1 h at r.t. Blots were probed with 1:5,000 primary antibody at 4 °C overnight. Blots were then washed with 1× PBS-T and probed with 1:5,000 secondary antibody for 1.5 h at r.t using an HRP-linked secondary antibody. Blots were washed with 1× PBS-T and exposed to ECL chemiluminescent substrate (Pierce ECL Plus Western Blocking Substrate, Thermo Scientific). Chemiluminescent signal was detected using the BioSpectrum AC Imaging system. Blots were probed with anti-H3K18cr (PTM-517) and anti-H3 (Invitrogen PA5-16183) primary antibodies.

### Fluorescence imaging

Imaging was performed with the Leica TCS SP8 STED 3X confocal microscope at ×40 and ×20 magnification. Regions of interest were selected on the basis of consistent cell density or consistent tissue morphology. For quantification of immunofluorescence images, raw files were analysed for mean fluorescence intensity (MFI) using ImageJ/Fiji (v.2.3.0). For in vitro analysis, 10+ random cells per image were outlined and their MFI was quantified. For in vivo analysis, 20–40 regions of interest were quantified per treatment group depending on availability in the tissue. For H3K18cr rhesus macaque tissue MFI analysis, 25 random regions containing crypts and villi per animal were imaged and analysed for fluorescent signal. ZO-1 continuity was calculated by dividing ZO-1 stain length by total epithelial length from the area selected.

### ROS fluorescence detection assay

Caco-2 cells (7 × 10^5^ per well) were seeded into 8-well chamber slides to reach 80% confluency. They were then treated with the previously described treatments for 24 h. Media were removed after treatment and cells were incubated in CellROX Green in cell culture media (1:300) (Thermo Fisher) for 1 h. Cells were then washed with PBS and fixed with 4% PFA for 20 min. Chamber slides were covered with Prolong Diamond Antifade mounting media and imaged immediately. The assay was run in duplicate.

### Quantitative real-time PCR

Total RNA was extracted from Caco-2, HCT-116 and SCDM cells using the QIAShredder (Qiagen), RNeasy mini kit (Qiagen) and RNA Clean and Concentrator (Zymo) kits according to manufacturer recommendations. DNA was removed with the RNA Clean and Concentrator kit using the supplied DNase I (Zymo). Samples were diluted to equal RNA concentration after measurement by NanoDrop. For complementary DNA (cDNA) synthesis, a master mix containing 1.5 μl Milli-Q water, 4 μl 5× buffer, 1 μl dNTP, 1 μl dithiothreitol, 0.25 μl RNA OUT and 0.25 μl SSIII was created per 15 μl sample of cleaned RNA. Samples were placed into a thermocycler and run at 50 °C for 180 min, 85 °C for 5 min and held at 4 °C. Samples were then diluted with 100 μl Milli-Q water. For RT-qPCR, the *Taq*man system was utilized (Thermo Fisher). For each well of a target gene, a master mix of 5 μl 2× *Taq*man Universal PCR Master Mix, 2.5 μl nuclease-free Milli-Q water and 0.5 μl *Taq*man Primer/Probe was created. RT-qPCR was run on the ViiA7 machine for 30 cycles. All positive signal was reported. Caco-2 and HCT-116 samples were run in triplicate and SCDM samples were run in duplicate. β-actin was used as housekeeping gene. Signals were reported as log_2_ fold change over untreated cells using the ΔΔ*C*_T_ method.

### Electron microscopy

Gut tissue was fixed in Karnovsky’s fixative (Electron Microscopy Sciences) immediately after removal from the animal and submitted to the NEI microscopy core at UC Davis for resin embedding and staining. Pieces (1 mm^3^) were cut and washed with Karnovsky’s fixative then fixed with 1% OsO_4_ in 0.1 M PO_4_ buffer for 2 h. Samples were then dehydrated using increasing concentrations of acetone washes. Samples were then embedded in epoxy resin. Sections averaging 70 nm were cut with a diamond knife (Diatome) and placed on copper grids. Sections were stained with uranyl acetate and lead citrate before being placed on an FEI Talos 120C transmission electron microscope. Digital images were captured with the FEI Ceta 4k × 4k CMOS camera.

### ChIP-seq

Chromatin immunoprecipitation was performed using the ChIP-IT express kit (Active Motif). Caco-2 cells were seeded on 175 cm^2^ flasks at a density of 3 × 10^6^ cells. Once at 90% confluency, cells were treated for 6 h with 10-HSA, NaCr or No Treatment, fixed with 11% formaldehyde and manually lysed with a Dounce homogenizer. Approximately 2 × 10^7^ cells were used per treatment. Sonication was conducted using the Covaris E220 focused ultrasonicator (Covaris) for 480 s at the UC Davis DNA Technologies Core. To precipitate chromatin associated with crotonyl binding, the ChIP-validated pan anti-crotonyllysine rabbit pAb antibody was used (PTM-501 PTM Biolabs). Chromatin (25 ng) was used per ChIP reaction. Library preparation was conducted with the KAPA HyperPrep kit (Roche Sequencing) and sequenced using the Illumina NextSeq 550 system PE40 with a Q30 > 87% (Illumina) at the UC Davis DNA Tech Core PE40 with 20 million reads per sample. Sequence was mapped to human genome hg38. To determine significantly enriched regions of the genome in each treatment, peak calling using Genrich was conducted against input DNA control with usegalaxy.org using FDR < 0.2 and *P* < 0.05 for peak calling. The Genomic Regions Enrichment of Annotations Tool (GREAT) web application from Stanford was used to determine genes associated with each peak. GREAT was used to determine significantly enriched (*P* < 0.05) biological and molecular pathways. HOMER was used to identify enriched transcription factor binding sites for each group. The ChIPSeeker application in R was used to visualize genomewide called peaks and association with different elements of DNA.

### Flow cytometry

Flow cytometry was performed at the UC Davis Flow Core to determine J-Lat HIV LTR activation after treatment with 10-HSA or sodium crotonate or PMA for 18 h. Cells were washed with 1× PBS and dyed with DAPI to determine viability, and gates were created to isolate live GFP-positive cell populations from each group. Analysis was conducted on FlowJo 10.8.2. Treatments were run in triplicate. Flow cytometric analysis of macaque lamina propria lymphocytes was performed to determine the distribution of T cells in jejunal lamina propria lymphocytes (LPL) samples at the CNPRC Flow Core. Cells were isolated from LPLs at necropsy timepoint and washed with 1× PBS. Cells were incubated with pre-conjugated antibody mix (Live/Dead Aqua, CD45, CD3, CD4, CD8, HLA-DR) for 30 min at 4 °C. Cells were washed and fixed with 4% PFA. Dead cells were excluded from each analysis.

### Seahorse OCR

Cells were seeded at 150,000 per well in the XFe 96-well cell culture plate (Agilent) in growth media. At 6 h before analysis, media were changed to MEM with 5 mM glucose, 2 mM pyruvate and 1 mM glutamine containing no phenol red or sodium bicarbonate along with viral antigens and 10-HSA. Three replicates were used per timepoint per treatment. For the PPARα inhibitor assay, 30 min before analysis, cells were treated with 10-HSA, viral antigens and GW6471. HCT-116 and HCT-116^PPARαKO^ cells were seeded at 150,000 per well and allowed to adhere for 24 h. At 30 min before analysis, cells were treated with 10-HSA and viral antigens. OCR (pmol min^−1^) was recorded over 75 min with addition of 2.5 μM oligomycin, 2 μM carbonyl cyanide-p-(trifluoromethoxy)phenylhydrazone (FCCP) and 0.5 μM rotenone/antimycin A according to manufacturer specifications.

### TEER

Caco-2 cells were seeded in the Transwell Permeable Support 6.5 mm insert plate (Costar) and treated with 10-HSA and viral antigens for 24 and 48 h. Treatments were removed and cell culture media were placed in all wells. Electrical resistance was immediately tested using a Millicell ERS-2 Voltohmmeter with the MERSSTX01 Electrode (Millipore Sigma). The experiment was run in triplicate.

### Luciferase reporter gene assay

Assays for determining the activation of PPARα were performed by Indigo Biosciences. Reporter CHO cells used is this study express either the native receptor or a receptor hybrid in which the native N-terminal DNA binding domain (DBD) has been replaced with that of the yeast Gal4 DBD. The reporter gene, firefly luciferase, is functionally linked to upstream receptor-specific genetic response elements or the Gal4 upstream activation sequence. A suspension of reporter cells was prepared in INDIGO’s cell recovery medium containing 10% charcoal-stripped FBS. Reporter cell suspension (100 μl) was dispensed into wells of a white 96-well assay plate and treated with 10-HSA (1.92 nM, 9.60 nM, 48.0 nM, 240 nM, 1.2 μM, 6 μM and 30 μM) for 24 h. Following the incubation period, treatment media were discarded and 100 μl of Luciferase Detection Reagent was added per well to determine receptor activity in terms of relative luminescence units. Receptor agonist GW7647 was used as PPARα positive control. Media control (with 0.1% DMSO) served as negative control.

### 10-HSA quantity in ileal contents

Mass spectrometry was performed by Metabolon using ultra high-performance liquid chromatography/tandem accurate mass spectrometry (UHPLC/MS/MS) using ileal contents of rhesus macaques treated with *L. plantarum* to determine relative metabolite concentrations. Data normalization and processing was conducted using the raw peak values from Metabolon using the MetaboAnalyst one-factor statistical analysis software pipeline. Untargeted metabolomic profiling of peripheral blood was performed by Metabolon. Serum (500 μl) isolated from peripheral blood from each animal in each group (SIVpos, SIVpos-HSA, SIVpos + ART, *n* = 3 per group) was submitted. To evaluate the effects of 10-HSA treatment, SIVpos-HSA, SIVpos and SIVpos + ART groups were normalized together, and partial least squares discriminant analysis (PLS-DA) and bar charts were created on the basis of significantly altered metabolites (*P* < 0.05). Data normalization and processing was conducted using the raw peak values from Metabolon using the MetaboAnalyst one-factor statistical analysis software pipeline.

### Gut microbiome 16S and metagenomic sequencing and analysis

Colo-rectal swabs were taken at necropsy and total DNA was isolated using the MoBio Power Soil kit (Qiagen) with one modification. After addition of CD1 buffer, samples were incubated at 65 °C for 10 min and then homogenized with the Qiagen Tissue Lyser II (Qiagen) and eluted in 100 μl buffer C6. The V3–V4 domain was amplified by PCR using the 341F and 806R primers. Each 25 μl PCR reaction contained 1 unit Kapa2G Robust Hot Start Polymerase, 1.5 mM MgCl_2_, 0.2 mM dNTP, 0.2 μM primers, 3 μl KAPA 5× Enhancer 1 and 1 μl DNA. PCR conditions were: an initial incubation at 95 °C for 3 min, followed by 25 cycles of 95 °C for 45 s, 50 °C for 30 s, 72 °C for 30 s and a final extension of 72 °C for 3 min. Then libraries were pooled and checked for quality and proper amplicon size on the Agilent 2100 Bioanalyzer at the UC Davis DNA Technologies Core. The libraries were then sequenced at 300PE on an Illumina Miseq at the UC Davis DNA Technologies Core. QIIME2 analysis was utilized to obtain Bray–Curtis beta diversity and Shannon alpha diversity data. LEfSe analysis of level 7 QIIME2 taxonomic data was used to generate cladogram and LDA plots (*a* < 0.15, *w* < 0.05, LDA > 2). Analysis was performed again in R to validate QIIME2 and LEfSe approaches with DEseq2. Demultiplexed and trimmed 16S sequences were denoised with the DADA2 (v.1.36.0) pipeline and assigned taxonomy using the SILVA 138.2 reference database. Phyloseq (v.1.52.0), vegan (v.2.7.2) and FSA (v.0.10.0) were used to visualize and analyse alpha diversity metrics such as Chao1, Shannon and Simpson, and Bray–Curtis beta diversity metrics. Differential abundance analyses were performed with DESeq2 (v.1.48.2) at an FDR < 0.05 significance value and were visualized with ggplot2 (v.4.0.0). Metagenomic library prep and sequencing was conducted by the UC Davis DNA Technologies Core. Low-quality bases, reads and sequencing adaptors were filtered from paired-end shotgun metagenomic reads using cutadapt (v.2.6, minimum-length: 50 max-*n*: 1 q: 30). Quality controlled reads were then aligned to the rhesus macaque genome (Mmul_10: GCA_003339765.3) with bowtie2 (v.2.5.1, sensitive end-to-end alignment) and unaligned reads were input into HUMAnN3 (v.3.0.1) for taxonomic and functional profiling. Resulting gene abundance tables were normalized (counts per million), joined and used for downstream analysis. LEfSe analysis was performed on metagenomic data to identify significantly (*a* < 0.15, *w* < 0.05, LDA > 2) altered taxa and pathways. Limma.voom was utilized to identify differential counts of microbial enzyme genes with significance cut-off of FDR < 0.15 and *P* < 0.05.

### GaMD simulations

The structural template for PPARα was retrieved from the Protein Data Bank (PDB) identifier 6KAX ref. ^51^. Any incomplete regions, including missing residues and heavy atoms, were reconstructed using Modeller 10.4. For the ligands, interaction parameters were defined following the general AMBER force field (GAFF2), with parameters refined through CHARMM-GUI^52–54^. The simulations used the optimal point change (OPC) water model and physiological concentrations of NaCl. To balance the total system charge, two Na+ counter ions were added. Protein residues were modelled using the FF19SB force field^55^. The PPARα–10-HSA complexes were subjected to an initial energy minimization employing 2,500 steps each of steepest descent and conjugate gradient. Following this, each system was gradually heated from 0 to 300 K over a 30 ps interval under constant volume using a Langevin thermostat with 1 fs timestep and a collision frequency of 2 ps^−1^. Initial velocities were assigned on the basis of the Maxwell–Boltzmann distribution. Throughout this phase, weak restraints were maintained on the heavy atoms of both the solute and the ligand, with the restraint strength methodically reduced from 100 kcal mol^−1^ Å^−2^ to 10, then to 1, and finally to 0.1 kcal mol^−1^ Å^−2^ before being completely removed. This progressive reduction facilitated the relaxation of the system, preparing it for subsequent steps without restricted molecular movement. Following the heating phase, each system underwent equilibration in the isothermal–isobaric (NPT) ensemble at a constant temperature of 310 K and pressure of 1 atm. Pressure was regulated using an isotropic Berendsen barostat with a relaxation time of 2 ps, ensuring smooth pressure adjustments. Non-bonded interactions were handled with a 10 Å cut-off, and long-range electrostatic interactions were computed using the particle mesh Ewald (PME) method to accurately represent electrostatic forces across the simulation box. In addition, the SHAKE algorithm was employed to constrain all hydrogen bonds to their equilibrium lengths, maintaining structural integrity while allowing other atomic movements. We implemented GaMD simulations using the GaMD module available in the GPU-enhanced AMBER22 software. Production simulations were facilitated by integrating Newton’s equations with a timestep of 2 fs. Initially, our systems were equilibrated for 100 ns within the NPT ensemble following protocols similar to those previously mentioned, albeit without applying any restraints. This phase was followed by a 65-ns period where a boost potential was introduced, serving to gather acceleration potential parameters essential for GaMD. Subsequently, five independent runs were performed in the NVT ensemble. During these production simulations, initial atomic velocities were randomized to ensure sampling. For these simulations, a dual-boost approach was employed. This involved applying one boost potential to the dihedral energy term and another to the total potential energy term, with the threshold energy set to the lower bound, *E* = *V*_max_. We recorded the average and standard deviation (s.d.) of the potential energies at every 200,000 steps, equivalent to 400 ps, setting the upper limit for the boost potential s.d., *σ*0, at 6.0 kcal mol^−1^ for both assessed energy terms. To visualize and analyse the trajectories from these GaMD simulations, we utilized a combination of software tools. VMD (v.1.9.4a57) and PyMol v.3.0 (Schrödinger) were employed for generating visual trajectory and interactions.

### 3’ RNA-seq, transcriptome alignment and pathway analysis

Total RNA was extracted from Caco-2, HCT-116 and SCDM cells using the QIAShredder (Qiagen), RNeasy mini kit (Qiagen) and RNA Clean and Concentrator (Zymo) kits according to manufacturer recommendations. DNA was removed with the RNA Clean and Concentrator kit using the supplied DNase I (Zymo). Samples were run on the Agilent 2100 Bioanalyzer at the UC Davis DNA Technologies Core to ensure that DNA contamination was removed from samples and RNA was the proper size for library preparation. Library preparation was performed using the QuantSeq 3’ mRNA-Seq V2 Library Prep kit with unique dual indexing (UDI). Samples were pooled with equal molarity, and sequencing was performed on an Evident AVITI sequencer (4 million reads per sample, *Q*_30_ > 95%). Samples were run through FastQC and STAR alignment on the Cancer Genomics Cloud using the Seven Bridges online platform. ‘Reads per gene’ tables were generated for each sample. The R package limma.voom was utilized for differential gene expression analysis between groups. Genes were assigned as significant (*P* ≤ 0.05 and FDR ≤ 0.15 for macaque RNA-seq, and *P* < 0.05 for SCDM RNA-seq) and utilized for downstream pathway analysis with metascape. Pathway enrichment was conducted using Metascape.org.

### Human study participants, sample collection and analysis

Five ART-naïve seropositive LTNP individuals, four HIV-negative and three HIV-positive high viral load patients were enrolled in this study. Parameters for each group are defined in the main text. Endoscopic jejunal biopsies were taken and cryo-preserved for immunofluorescence and microarray analysis. Total RNA was isolated from tissue samples using the RNeasy kit (Qiagen) and cDNA was synthesized (Superscript Choice system, Gibco) using an oligo(dT_24_) primer. Biotin-labelled cRNA was hybridized with Affymetrix U95Av2 Gene Chip. GeneChips were normalized using the dChip software. Genes differentially expressed in GALT were hierarchically clustered to identify patterns of up and downregulation in response to HIV infection and ART. The Institutional Review Board at UC, Davis approved the study (UCD IRB #244879-23), and written informed consent was obtained from all participants of the study. This study further analysed data underlying a previous publication^40^. Microarray analysis using sample ‘fold changes’ was conducted using LTNP (*n* = 3) and HVL (*n* = 4) from a previously published dataset^40^.

### X-ray crystallography, PPARα-LBD protein expression and purification

Construct for the expression of human PPARα-LBD (residues 200–468) in pET-24a(+) vector containing N-terminal His_6_- and Avi-tags, and a thrombin protease cleavage site was transformed into T7 Express cells (NEB). Transformed cells were cultured in TB medium supplemented with 50 µg ml^−1^ kanamycin, 5% w/v glucose, 5 mM MgCl_2_ and 0.2% w/v ammonium sulfate at 30 °C overnight. A starter culture was used to inoculate large-scale cultures in TB medium containing 50 µg ml^−1^ kanamycin, 0.4% w/v glucose, 5 mM MgCl_2_ and 0.2% w/v ammonium sulfate. The cultures were grown at 37 °C with shaking at 200 r.p.m. until growth reached optical density at 600 nm (OD_600_) of ~10, at which point the temperature was lowered to 18 °C and protein expression was induced with 1 mM isopropyl β-D-1-thiogalactopyranoside (IPTG). Bacterial pellets were resuspended in Ni-NTA buffer A (20 mM Tris (pH 8.0), 200 mM NaCl, 10% glycerol, 1 mM Tris(2-carboxyethyl)phosphine (TCEP)) containing EDTA-free Protease Inhibitor Cocktail (Abcam) and BaseMuncher endonuclease (Abcam). Cells were lysed in one pass in a cell disruptor (Constant Systems) at 38 kpsi. Cell lysate was clarified by centrifugation at 50,000 × *g* for 30 min at 4 °C. The resulting clarified lysate was loaded onto an Omnifit column prepacked with 3 ml Ni-NTA resin (Qiagen) pre-equilibrated in Ni-NTA buffer A. Following sample application, the column was washed with 20 column volumes (CVs) of Ni-NTA buffer A, followed by 5 CVs of high-salt Ni-NTA buffer B (20 mM Tris (pH 8.0), 500 mM NaCl, 10% glycerol, 1 mM TCEP) and an additional 10 CVs of Ni-NTA buffer A. To remove non-specifically bound proteins, the column was washed with 5 CVs of 2% Ni-NTA buffer C (20 mM Tris (pH 8.0), 200 mM NaCl, 400 mM imidazole, 10% glycerol, 1 mM TCEP). Bound protein was eluted by washing the column with 5 CVs of 25% buffer C (that is, 100 mM imidazole). To cleave off the affinity tag from the PPARα-LBD protein, the Ni-NTA pool was dialysed with thrombin protease (500 units) overnight in 5 l of Ni-NTA buffer A. Reverse Ni-NTA method was performed to remove the cleaved tag before the final polishing size-exclusion chromatography (SEC) step on the HiLoad 26/600 Superdex 75 pg gel filtration column (Cytiva) pre-equilibrated in SEC buffer (50 mM Tris (pH 8.0), 150 mM NaCl, 10% glycerol, 1 mM TCEP). Peak fractions from the SEC step were pooled, concentrated to 12.7 mg ml^−1^ and flash frozen in liquid nitrogen. The purity of PPARα-LBD protein was analysed at each purification stage using SDS–PAGE and Coomassie Blue staining (Extended Data Fig. 1c). For PPARα-LBD crystallization, initial crystallization trials with HSA did not yield hits in any condition. WY-14643 has previously been reported to co-crystallize with PPARα and seeds generated from these crystals were used by the authors to successfully obtain crystals with additional ligands that did not co-crystallize on their own^56^. In accordance with these methods, JCSG+ Eco (Molecular Dimensions) and Index (Hampton Research) sparse matrix screens were set up with PPARα at 10 mg ml^−1^ and 1 mM WY-14643, yielding crystals in multiple conditions. A seedstock was generated from crystals obtained from JCSG+ screen condition #E12 (0.1 M imidazole pH 8.0 + 10% PEG8000) by crushing the crystals and resuspending them as a 1:4,000 dilution with the #E12 precipitant. Additional JCSG+ Eco and Index screens were then set up with 10% v/v addition of the diluted seedstock. Sitting drops were set up by Mosquito robot: 200 nl PPARα at 10 mg ml^−1^ + 0.5 mM HSA, 180 nl well solution and 20 nl seedstock These screens yielded crystals with HSA in multiple conditions. Crystals were prepared for synchrotron testing by layering drops with 1.5 μl of cryoprotectant formed by mixing 20% v/v of 5 mM HSA in 1:1 DMSO:ethylene glycol with 80% v/v well solution. Crystals were incubated for ~5 min and collected using Mylar loops and vitrified by immersion in liquid nitrogen. Data were collected at Diamond Synchrotron, Beamline I03 (*λ* = 0.976 Å), using a Dectris Eiger2 XE 16 M detector. Datasets were indexed and integrated with Xia2 DIALS, scaled and merged with Aimless and phased by molecular replacement with Phaser using chain-A of PDB model 6LX6 (refs. ^57–59^). The 2F_o_-F_c_ maps contoured at 1*σ* showed unequivocal electron density for HSA. The resultant model was improved by iterative cycles of manual model building in Coot and refinement in Refmac^60,61^. The final model has 98.4% of residues in the most favoured regions of the Ramachandran plot and clash score of 3.7 as defined by MolProbity^8^. CCP4mg was utilized for visualization of the structure. This analysis was performed by Charles River Laboratories.

### Statistical analysis

Data shown represent the mean ± s.e.m calculated by using all data points unless otherwise stated. Statistical significance was determined using unpaired *t*-tests when comparing values between two groups. Analysis of variance (ANOVA) was used to determine treatment effects between multiple groups. *P* values < 0.05 were considered significant. All analysis was performed in GraphPad Prism (v.9.4.0).

### Reporting summary

Further information on research design is available in the Nature Portfolio Reporting Summary linked to this article.

## Supplementary information

Supplementary Information Supplementary Figs. 1–3 and Tables 1–4.

Reporting Summary

Supplementary Data Source data for Supplementary Fig. 1.

## Source data

Source Data Fig. 1 Statistical source data.

Source Data Fig. 2 Statistical source data.

Source Data Fig. 3 Statistical source data.

Source Data Fig. 4 Statistical source data.

Source Data Fig. 6 Statistical source data.

Source Data Extended Data Fig. 1 Statistical source data.

Source Data Extended Data Fig. 2 Statistical source data.

Source Data Extended Data Fig. 3 Statistical source data.

Source Data Extended Data Fig. 4 Statistical source data.

Source Data Extended Data Fig. 5 Statistical source data.

Source Data Extended Data Fig. 8 Statistical source data and unprocessed western blots.

Source Data Extended Data Fig. 10 Statistical source data.

## Data Availability

ChIP-seq (PRJNA1083621), macaque RNA-seq and 16S seq (PRJNA1133740), macaque metagenomics sequencing (PRJNA1150326), human stem cell-derived monolayer RNA-seq (PRJNA1133748), ACT-treated animals RNA-seq (PRJNA1347858) and 16S (PRJNA1347860) data generated in this study are available in the Sequence Read Archives (SRA) NCBI database. X-ray crystallography data are available on the Protein Data Bank (PDB) with ID 9SFS. Source data are provided with this paper.
