# Supplementary material for: Microbiota-derived 10-hydroxystearic acid activates PPARα to restore gut epithelial barrier integrity and enhance anti-retroviral therapy
Source: Nat Microbiol. 2026 Jul 30;11(8):2365–83. doi: 10.1038/s41564-026-02433-0 (PMC13423806; doi:10.1038/s41564-026-02433-0)
Supplement: Supplementary file 1 — Supplementary Figs. 1–3 and Tables 1–4. [file 41564_2026_2433_MOESM1_ESM.pdf]

nature microbiology

Supplementary information

https://doi.org/10.1038/s41564-026-02433-0

# Microbiota-derived 10-hydroxystearic acid activates PPARα to restore gut epithelial barrier integrity and enhance anti-retroviral therapy

In the format provided by the authors and unedited

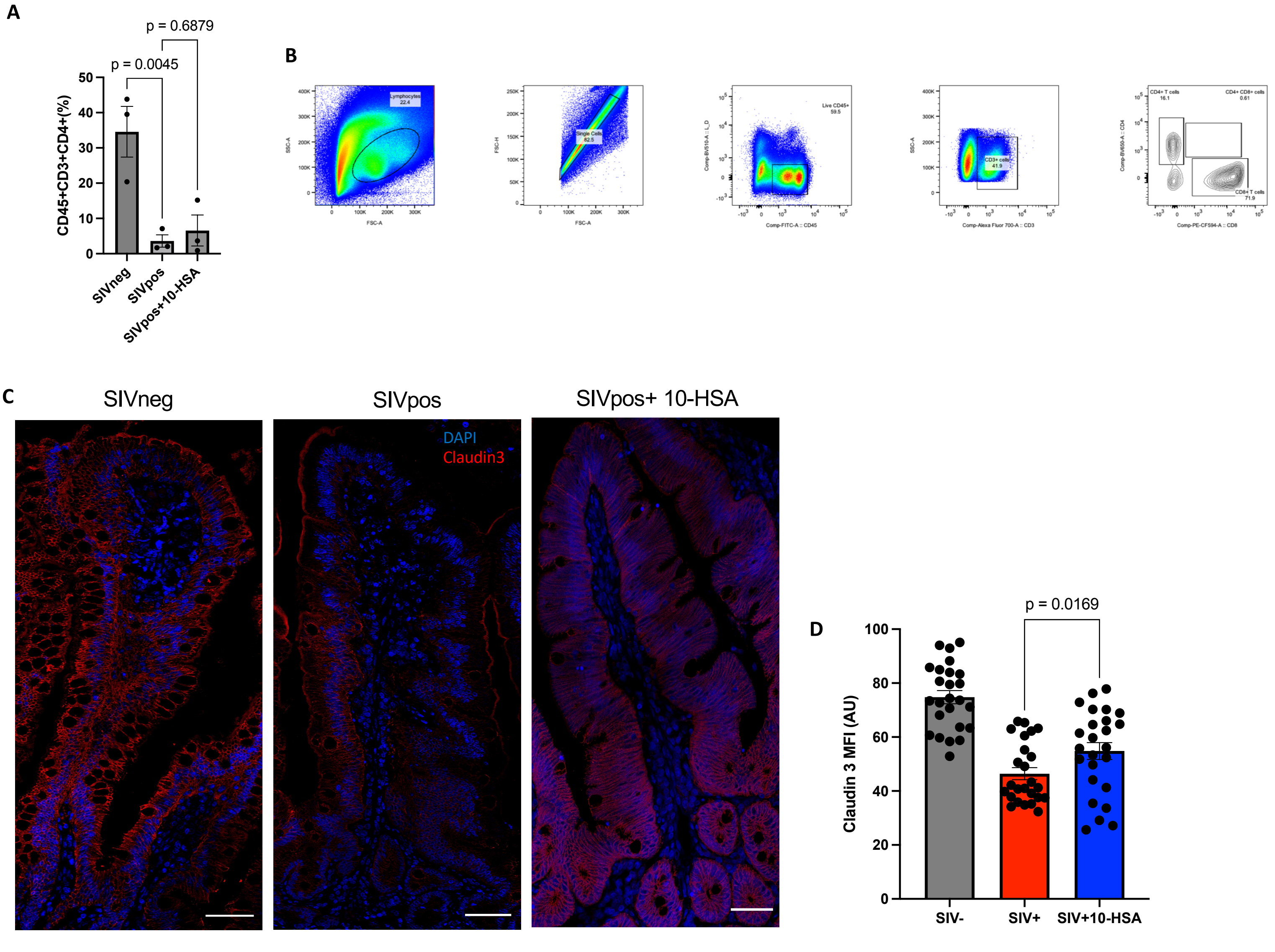


**Fig. 1. Treatment with 10-HSA promoted gut epithelial tight junction structure *in vivo*.** (A) Mucosal CD4+ T-cell percentages as determined by flow cytometry (n = 3 biological replicates per group). Data analyzed by one-way ANOVA. (B) Gating strategy. (C) Claudin-3 immunofluorescence in SIVneg, SIVpos, and SIVpos+10-HSA treated animals, (n = 3 biological replicates per group). (D) Semi-quantification of Claudin-3 signal by one-way t test. Data are presented as mean values +/- SEM. Scale bar = 50µm.

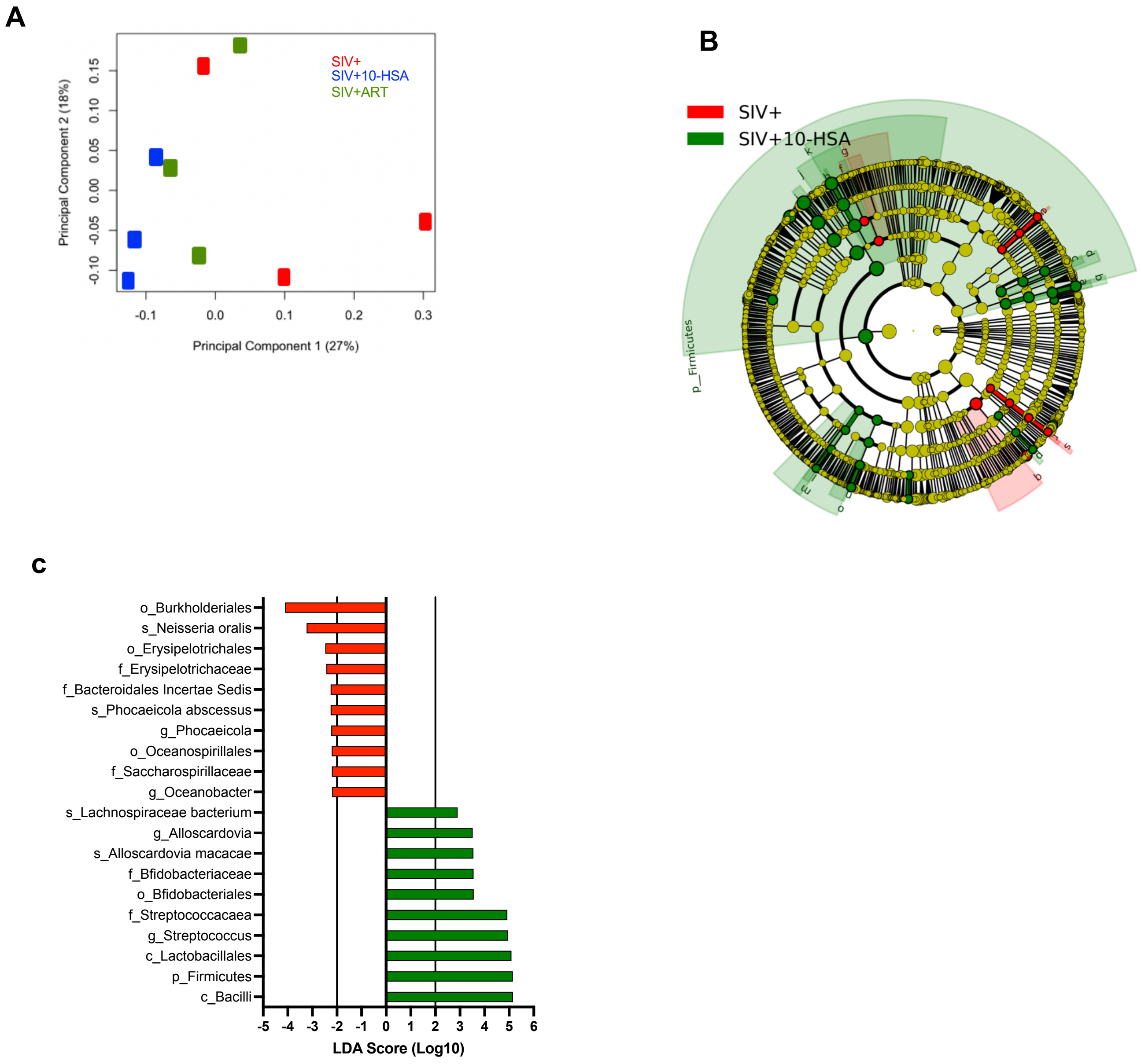


**Fig. 2. Increased populations of Firmicutes in the gut and oral mucosal sites following 10-HSA treatment.** (A) Bray-Curtis beta diversity metrics (n = 3 biological replicates per group). (B) LEfSe cladogram output comparing SIVpos and SIVpos+10-HSA oral microbiome compositions (n = 3 biological replicates per group). (C) LEfSe output showing enriched taxa in SIVPOS (red) and SIVPOS10-HSA (green) oral microbiomes (n = 3 biological replicates per group).

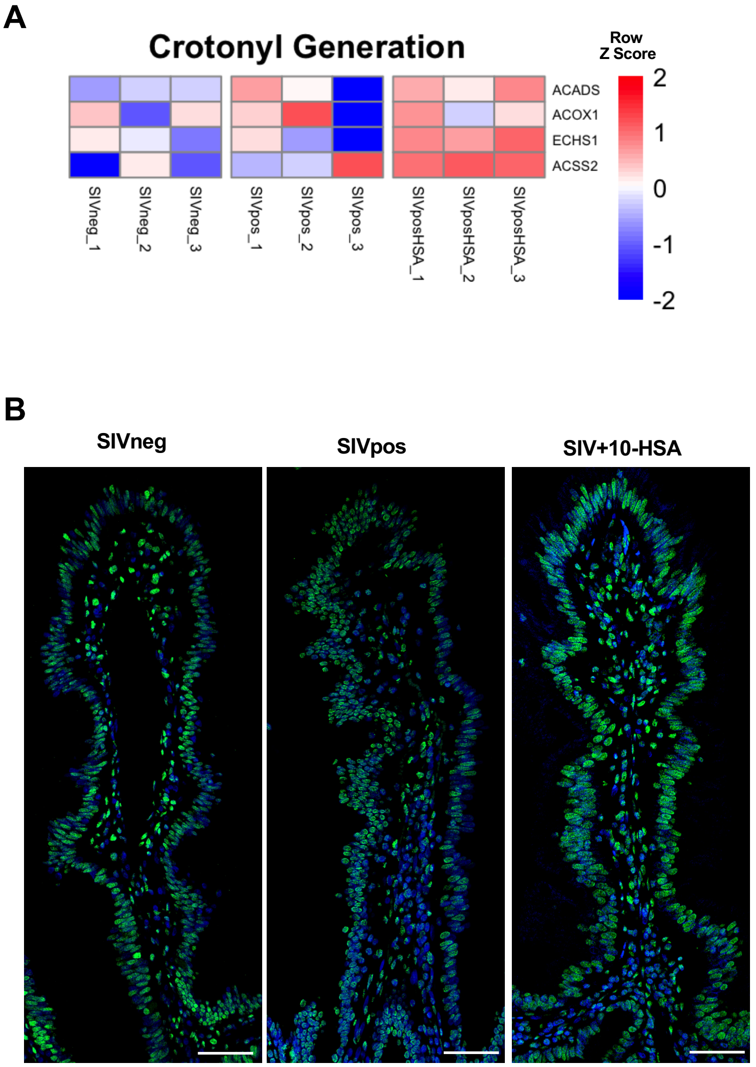


**Fig. 3. Increased expression of crucial crotonyl group producing enzymes following 10-HSA treatment.** (A) Expression of key crotonyl group producing enzymes in animals treated with 10-HSA (n = 3 biological replicates per group). (B) Histone acetylation representative images in SIVneg, SIVpos, and SIVpos+10-HSA treated animals (n = 3 biological replicates per group).

**Table 1. Top 5 upregulated metabolites due to LP treatment *in vivo*.**

| Biochemical | Super Pathway | PubChemID | FC LP+/LP- |
|---|---|---|---|
| 10-hydroxystearic acid | Lipid | 9561835 | 123 |
| N-acetylglutamine | Amino Acid | 182230 | 25 |
| Cytosine | Nucleotide | 597 | 22 |
| Phosphoenolpyruvate | Carbohydrate | 1005 | 16 |
| Nicotinamide adenine dinucleotide | Cofactors/Vitamins | 5893 | 15 |

**Table 2. X-ray Crystallography table of statistics.**

| | hPPARα-HSA |
|---|---|
| **Data collection** | |
| Space group | P 1 21 1 |
| Cell dimensions | |
| *a*, *b*, *c* (Å) | 44.56, 61.25, 53.08 |
| α, β, γ (°) | 90.00, 106.86, 90.00 |
| Resolution (Å) | 61.25-2.0 (2.05-2.0)* |
| $R_{sym}$ or $R_{merge}$ | 0.222 (0.86) |
| $I / \sigma I$ | 4.7 (1.6) |
| Completeness (%) | 99.5 (99.6) |
| Redundancy | 5.2 (5.1) |
| **Refinement** | |
| Resolution (Å) | 50.80-2.1 |
| No. reflections | 17,600 |
| $R_{work}$ / $R_{free}$ | 0.2305/0.2803 |
| No. atoms | |
| Protein | 2,086 |
| Ligand/ion | 25 |
| Water | 67 |
| *B*-factors | |
| Protein | 24.5 |
| Ligand/ion | 28.0 |
| Water | 23.4 |
| R.m.s. deviations | |
| Bond lengths (Å) | 0.009 |
| Bond angles (°) | 1.85 |

*Values in parentheses are for highest-resolution shell.

[AU: Equations defining various *R*-values are standard and hence are no longer defined in the footnotes.]
[AU: Ramachandran statistics should be in Methods section at the end of Refinement subsection.]
[AU: Wavelength of data collection, temperature and beamline should all be in Methods section.]

**Table 3. Human sample metadata**

| Microarray | | | | | | |
|---|---|---|---|---|---|---|
| Study Group | Patient ID | Age (years) | Sex | CDC classification | Year of HIV Diagnosis | Peripheral CD4+ T cell counts/mL |
| HVL | P13 | 48 | F | C3 | Unknown | 8 |
| HVL | P14 | 36 | M | C3 | 2001 | 6 |
| HVL | P19 | 35 | M | C3 | 2001 | 46 |
| HVL | P47 | 23 | M | C2 | 2002 | 205 |
| LTNP | P16 | 49 | M | A1 | 1989 | 910 |
| LTNP | P30 | 43 | M | A1 | 1997 | 587 |
| LTNP | P46 | 45 | M | A1 | 1985 | 787 |

| Immunofluorescence | | | |
|---|---|---|---|
| Study Group | Patient ID | Sex | Species |
| HIV- | 100 | Male | Human |
| HIV- | 101 | Male | Human |
| HIV- | 168 | Male | Human |
| HIV- | 131 | Male | Human |
| HVL | 149 | Male | Human |
| HVL | 146 | Male | Human |
| HVL | 170 | Male | Human |
| LTNP | 129 | Male | Human |
| LTNP | 96 | Male | Human |
| LTNP | 159 | Male | Human |
| LTNP | 93 | Male | Human |
| LTNP | 41 | Male | Human |

## Table 4. Primers and antibodies used in study.

| | | | | | | | |
|---|---|---|---|---|---|---|---|
| Antibody (Clone) | Fluorescence | Assay | Vendor Catalogue # | | | Primer Target | Catalogue Number |
| L/D Fixable Aqua | 405nm | Flow Cytometry | Life Tech L34957 | | | ACOX1 | HS01074241_M1 |
| CD45 MouseαNHP (D058-1283) | BV605 | Flow Cytometry | BD 564098 | | | ACADS | HS00163506_M1 |
| CD3 MouseαHuman (SP34-2) | AF700 | Flow Cytometry | BD 561805 | | | ACOX3 | Hs01089970_m1 |
| CD4 MouseαHuman (L200) | BV650 | Flow Cytometry | BD 563737 | | | ZO1 | Hs01551871_m1 |
| CD8 MouseαHuman (SK1) | PE-DAZZLE 594 | Flow Cytometry | Biolegend 344744 | | | Claudin1 | Hs00221623_m1 |
| HLA-DR MouseαHuman (L243) | PE-CY7 | Flow Cytometry | Biolegend 307615 | | | Claudin3 | Hs00265816_s1 |
| ZO-1 Mouse monoclonal | - | IF Primary | Invitrogen 33-9100 | | | ACSS2 | Hs01122829_m1 |
| H3K18cr RabbitαHuman | - | IF Primary | PTM-517 | | | ACADM | HS00936584_M1 |
| H3K18ac RabbitαHuman | - | IF Primary | Invitrogen PA5-85523 | | | ACADVL | HS00825606_G1 |
| H3K14ac MouseαHuman | - | IF Primary | AM 61433 | | | ACSL3 | HS00244853_M1 |
| Pan-crotonyl-lysine RabbitαHuman | - | ChIP | PTM-501 | | | HADHA | HS00426191_M1 |
| AF488 GoatαRabbit | 488 | IF Secondary | Thermo A11034 | | | ECHS1 | HS00187943_m1 |
| AF555 GoatαMouse | 555 | IF Secondary | Thermo A21422 | | | | |
| AF647 GoatαMouse | 647 | IF Secondary | Thermo A21235 | | | | |
