# Supplementary material for: Microbiota-derived 10-hydroxystearic acid activates PPARα to restore gut epithelial barrier integrity and enhance anti-retroviral therapy
Source: Nat Microbiol. 2026 Jul 30;11(8):2365–83. doi: 10.1038/s41564-026-02433-0 (PMC13423806; doi:10.1038/s41564-026-02433-0)
Supplement: Supplementary file 2 — Reporting Summary [file 41564_2026_2433_MOESM2_ESM.pdf]

nature portfolio

Corresponding author(s): Satya Dandekar

Last updated by author(s): 5/27/2026

Reporting Summary

Nature Portfolio wishes to improve the reproducibility of the work that we publish. This form provides structure for consistency and transparency in reporting. For further information on Nature Portfolio policies, see our Editorial Policies and the Editorial Policy Checklist.

Statistics

For all statistical analyses, confirm that the following items are present in the figure legend, table legend, main text, or Methods section.

n/a	Confirmed	
☐	☒	The exact sample size (*n*) for each experimental group/condition, given as a discrete number and unit of measurement
☐	☒	A statement on whether measurements were taken from distinct samples or whether the same sample was measured repeatedly
☐	☒	The statistical test(s) used AND whether they are one- or two-sided<br>*Only common tests should be described solely by name; describe more complex techniques in the Methods section.*
☐	☒	A description of all covariates tested
☐	☒	A description of any assumptions or corrections, such as tests of normality and adjustment for multiple comparisons
☐	☒	A full description of the statistical parameters including central tendency (e.g. means) or other basic estimates (e.g. regression coefficient) AND variation (e.g. standard deviation) or associated estimates of uncertainty (e.g. confidence intervals)
☐	☒	For null hypothesis testing, the test statistic (e.g. F, t, r) with confidence intervals, effect sizes, degrees of freedom and P value noted<br>*Give P values as exact values whenever suitable.*
☒	☐	For Bayesian analysis, information on the choice of priors and Markov chain Monte Carlo settings
☒	☐	For hierarchical and complex designs, identification of the appropriate level for tests and full reporting of outcomes
☐	☒	Estimates of effect sizes (e.g. Cohen's *d*, Pearson's *r*), indicating how they were calculated

Our web collection on statistics for biologists contains articles on many of the points above.

Software and code

Policy information about availability of computer code

Data collection	Sequencing data was collected with Element Aviti and Illumina NextSeq 550, X-ray crystallography data was collected with Diamond Synchrotron Dectris Eiger2 XE 16M detector.
Data analysis	Prism 10.0.0 was used for data analysis for in vitro and ex vivo and in vivo experiments. RNA sequencing was analyzed using limma.voom in RStudio (v2023.06.0). ChIP-seq data utilized FASTQC, Bowtie2, SAMtools, Genrich, GREAT, HOMER, ChIPSeeker. Metagenomics data was analyzed with bowtie2, HUMAnN3. 16S and Metagenomics data were also analyzed with DEseq2 and LEfSe. GaMD simulation included use of Modeller 10.4, CHARMM-GUI, Particle Mesh Ewald, SHAKE, AMBER22, VMD v1.9.4a57, PyMol Version 3.0. X-ray crystallography analysis utilized Coot, Refmac, MolProbity, and CCP4mg. Flow cytometry data was analyzed with FlowJo.

For manuscripts utilizing custom algorithms or software that are central to the research but not yet described in published literature, software must be made available to editors and reviewers. We strongly encourage code deposition in a community repository (e.g. GitHub). See the Nature Portfolio guidelines for submitting code & software for further information.

Data

Policy information about availability of data

All manuscripts must include a data availability statement. This statement should provide the following information, where applicable:

- Accession codes, unique identifiers, or web links for publicly available datasets
- A description of any restrictions on data availability
- For clinical datasets or third party data, please ensure that the statement adheres to our policy

ChIP-seq (PRJNA1083621), macaque RNAseq and 16S seq (PRJNA1133740), macaque metagenomics sequencing (PRJNA1150326), human stem cell derived monolayer RNAseq (PRJNA1133748), ACT treated animals RNAseq (PRJNA1347858) and 16S (PRJNA1347860) data generated for this study is available in the Sequence Read Archives (SRA) NCBI database. X-ray crystallography data is available on Protein Data Bank (PDB) ID: 9SFS.

Research involving human participants, their data, or biological material

Policy information about studies with human participants or human data. See also policy information about sex, gender (identity/presentation), and sexual orientation and race, ethnicity and racism.

Reporting on sex and gender	N/A
Reporting on race, ethnicity, or other socially relevant groupings	N/A
Population characteristics	N/A
Recruitment	High viral load (HVL) individuals, Long term non-progressors (LTNP) and HIV negative (HIVneg) individuals were used for this study.
Ethics oversight	UC Davis IRB

Note that full information on the approval of the study protocol must also be provided in the manuscript.

Field-specific reporting

Please select the one below that is the best fit for your research. If you are not sure, read the appropriate sections before making your selection.

☒ Life sciences ☐ Behavioural & social sciences ☐ Ecological, evolutionary & environmental sciences

For a reference copy of the document with all sections, see nature.com/documents/nr-reporting-summary-flat.pdf

Life sciences study design

All studies must disclose on these points even when the disclosure is negative.

Sample size	Sample size was determined based on non-human primate availability.
Data exclusions	No data was excluded from analysis
Replication	Replication was conducted through multiple independent experimental runs.
Randomization	Animals were randomly assigned treatment groups
Blinding	Blinding during study and data analysis was conducted

Reporting for specific materials, systems and methods

We require information from authors about some types of materials, experimental systems and methods used in many studies. Here, indicate whether each material, system or method listed is relevant to your study. If you are not sure if a list item applies to your research, read the appropriate section before selecting a response.

Materials & experimental systems

n/a	Involved in the study
☐	☒ Antibodies
☐	☒ Eukaryotic cell lines
☒	☐ Palaeontology and archaeology
☐	☒ Animals and other organisms
☒	☐ Clinical data
☒	☐ Dual use research of concern
☒	☐ Plants

Methods

n/a	Involved in the study
☐	☒ ChIP-seq
☐	☒ Flow cytometry
☒	☐ MRI-based neuroimaging

Antibodies

Antibodies used

CD45 MouseαNHP (D058-1283) BD 564098
CD3 MouseαHuman (SP34-2) BD 561805
CD4 MouseαHuman (L200) BD 563737
CD8 MouseαHuman (SK1) Biolegend 344744
HLA-DR MouseαHuman (L243) Biolegend 307615
ZO-1 Mouse monoclonal Invitrogen 33-9100
H3K18cr RabbitαHuman PTM-517
H3K18ac RabbitαHuman Invitrogen PA5-85523
H3K14ac MouseαHuman AM 61433
Pan-crotonyl-lysine RabbitαHuman PTM-501
AF488 GoatantiRabbit Thermo A11034
AF555 Goat anti Mouse Thermo A21422
AF647 GoatantiMouse Thermo A21235

Primary antibodies 1:200 dilution, secondary antibodies 1:400 for IHC

Validation

Flow cytometry antibodies were previously validated - DOI: 10.1172/jci.insight.149033
ZO-1 antibody was previously validated - DOI: 10.1073/pnas.1908977116

All other antibodies were validated by manufacturer

Eukaryotic cell lines

Policy information about cell lines and Sex and Gender in Research

Cell line source(s)	ATCC HTB-37 Caco2, Cellosaurus J-lat 10.6, HCT-116 WT and PPARa KO
Authentication	https://www.atcc.org/products/htb-37, https://www.cellosaurus.org/CVCL_8281, https://www.ubigene.us/product/KO-Cell-Line/PPARA-Knockout-cell-line-HCT-116
Mycoplasma contamination	Not detected by ATCC, https://www.atcc.org/products/htb-37. J-lat 10.6 not tested by Cellosaurus https://www.cellosaurus.org/CVCL_8281, HCT-116 WT and PPARA KO are negative for Mycoplasma as determined by manufacturer: https://www.ubigene.us/product/KO-Cell-Line/PPARA-Knockout-cell-line-HCT-116
Commonly misidentified lines (See ICLAC register)	None

Animals and other research organisms

Policy information about studies involving animals; ARRIVE guidelines recommended for reporting animal research, and Sex and Gender in Research

Laboratory animals	Adult rhesus macaques housed in CNPRC
Wild animals	N/A
Reporting on sex	Sex was not considered for these studies. Both male and female non-human primates were enrolled.
Field-collected samples	N/A
Ethics oversight	Animal use and study design was approved by UC Davis IACUC board

Note that full information on the approval of the study protocol must also be provided in the manuscript.

Plants

Seed stocks	*Report on the source of all seed stocks or other plant material used. If applicable, state the seed stock centre and catalogue number. If plant specimens were collected from the field, describe the collection location, date and sampling procedures.*
Novel plant genotypes	*Describe the methods by which all novel plant genotypes were produced. This includes those generated by transgenic approaches, gene editing, chemical/radiation-based mutagenesis and hybridization. For transgenic lines, describe the transformation method, the number of independent lines analyzed and the generation upon which experiments were performed. For gene-edited lines, describe the editor used, the endogenous sequence targeted for editing, the targeting guide RNA sequence (if applicable) and how the editor was applied.*
Authentication	*Describe any authentication procedures for each seed stock used or novel genotype generated. Describe any experiments used to assess the effect of a mutation and, where applicable, how potential secondary effects (e.g. second site T-DNA insertions, mosiacism, off-target gene editing) were examined.*

ChIP-seq

Data deposition

☒ Confirm that both raw and final processed data have been deposited in a public database such as GEO.

☒ Confirm that you have deposited or provided access to graph files (e.g. BED files) for the called peaks.

Data access links<br>*May remain private before publication.*	PRJNA1083621
Files in database submission	Raw data files
Genome browser session<br>(e.g. UCSC)	N/A

Methodology

Replicates	Treatments consisted of 20 million Caco2 cells each to supply adequate size for analysis of DNA associated with histone crotonylation resulting from 10-HSA, Sodium Crotonate, and no treatment
Sequencing depth	40 million reads per sample
Antibodies	Pan Anti-crotonyllisine PTM-501
Peak calling parameters	GenRich peak caller was used with the following parameters from usegalaxy.org: Maximum q value - 0.05, minimum AUC per peak -20, Maximum distance between significant sites - 100
Data quality	Sequencing reads Q30 > 87.1%
Software	NextSeq 550 Illumina platform sequencer was used. BowTie2, SAMtools, BEDtools, GenRich, GREAT by Stanford

Flow Cytometry

Plots

Confirm that:

☒ The axis labels state the marker and fluorochrome used (e.g. CD4-FITC).

☒ The axis scales are clearly visible. Include numbers along axes only for bottom left plot of group (a 'group' is an analysis of identical markers).

☒ All plots are contour plots with outliers or pseudocolor plots.

☒ A numerical value for number of cells or percentage (with statistics) is provided.

Methodology

Sample preparation	Cells were isolated from LPLs and PBMCs at and washed with 1xPBS. Cells were incubated with Live/Dead Aqua, anti-CD45, anti-CD3, anti-CD4, anti-CD8, anti-HLA-DR antibodies for 1 hour. Samples were washed and fixed in 2% PFA overnight. Flow was run the next morning.
Instrument	BD FACSymphony A3
Software	FlowJo 10.8.2

Cell population abundance	Determined by FlowJo gating
Gating strategy	Shown in supplemental data

☒ Tick this box to confirm that a figure exemplifying the gating strategy is provided in the Supplementary Information.
